# Neuroimmunological Blood Brain Barrier Opening in Experimental Cerebral Malaria

**DOI:** 10.1371/journal.ppat.1002982

**Published:** 2012-10-25

**Authors:** Adela Nacer, Alexandru Movila, Kerstin Baer, Sebastian A. Mikolajczak, Stefan H. I. Kappe, Ute Frevert

**Affiliations:** 1 Division of Medical Parasitology, Department of Microbiology, New York University School of Medicine, New York, New York, United States of America; 2 Seattle Biomedical Research Institute, Seattle, Washington, United States of America; London School of Hygiene and Tropical Medicine, United Kingdom

## Abstract

*Plasmodium falciparum* malaria is responsible for nearly one million annual deaths worldwide. Because of the difficulty in monitoring the pathogenesis of cerebral malaria in humans, we conducted a study in various mouse models to better understand disease progression in experimental cerebral malaria (ECM). We compared the effect on the integrity of the blood brain barrier (BBB) and the histopathology of the brain of *P. berghei* ANKA, a known ECM model, *P. berghei* NK65, generally thought not to induce ECM, *P. yoelii* 17XL, originally reported to induce human cerebral malaria-like histopathology, and *P. yoelii* YM. As expected, *P. berghei* ANKA infection caused neurological signs, cerebral hemorrhages, and BBB dysfunction in CBA/CaJ and Swiss Webster mice, while Balb/c and A/J mice were resistant. Surprisingly, PbNK induced ECM in CBA/CaJ mice, while all other mice were resistant. *P. yoelii* 17XL and *P. yoelii* YM caused lethal hyperparasitemia in all mouse strains; histopathological alterations, BBB dysfunction, or neurological signs were not observed. Intravital imaging revealed that infected erythrocytes containing mature parasites passed slowly through capillaries making intimate contact with the endothelium, but did not arrest. Except for relatively rare microhemorrhages, mice with ECM presented no obvious histopathological alterations that would explain the widespread disruption of the BBB. Intravital imaging did reveal, however, that postcapillary venules, but not capillaries or arterioles, from mice with ECM, but not hyperparasitemia, exhibit platelet marginalization, extravascular fibrin deposition, CD14 expression, and extensive vascular leakage. Blockage of LFA-1 mediated cellular interactions prevented leukocyte adhesion, vascular leakage, neurological signs, and death from ECM. The endothelial barrier-stabilizing mediators imatinib and FTY720 inhibited vascular leakage and neurological signs and prolonged survival to ECM. Thus, it appears that neurological signs and coma in ECM are due to regulated opening of paracellular-junctional and transcellular-vesicular fluid transport pathways at the neuroimmunological BBB.

## Author Summary

*Plasmodium falciparum*, the deadliest of all human malaria parasites, can cause cerebral malaria, a severe and frequently fatal complication of this devastating disease. Young children are predominantly at risk and may progress rapidly from the first signs of neurological involvement to coma and death. Here we used a murine model for high-resolution *in vivo* imaging to demonstrate that cerebral malaria, but not high parasitemia and severe anemia, is associated with extensive leakage of fluid from cerebral blood vessels into the brain tissue. This vascular leakage occurs downstream from the capillary bed, at the neuroimmunological blood brain barrier, a site recently recognized as the immune cell entry point into the brain during neuroinflammation. Vascular leakage is closely associated with the appearance of neurological signs suggesting that the ultimate cause of brain edema, coma and death in cerebral malaria is a widespread opening of the neuroimmunological blood brain barrier. Indeed, vascular leakage, neurological signs, and death from ECM can be prevented with endothelial barrier-stabilizing drugs. Based on the unique role of this anatomical feature in neuroinflammation, our findings are expected to have implications for other infectious diseases and autoimmune disorders of the central nervous system.

## Introduction

Human cerebral malaria (HCM) is a serious neurological complication that occurs in about 1% of *P. falciparum* infections. Although the proportion of patients that develop HCM is relatively small, the total death toll in children under 5 years of age is still unacceptably high [1]–[4]. Even with optimal medical care, the mortality rate in comatose pediatric patients is 15–20%. HCM is characterized by a rapid progression from headache, general malaise, and prostration to hemiparesis, ataxia, unrousable coma, and death [3]. Pediatric HCM fatalities are most commonly due to respiratory arrest [5], but identifying the underlying pathology leading to death from HCM has proven difficult. Fatal outcome may require a combination of predisposing factors to transpire simultaneously, which could explain the low incidence of HCM mortality. Alternatively, death may be due to parasite-mediated injury to a sensitive location, for example in the brain stem, where a small lesion can cause sudden respiratory arrest [5], but evidence for this is lacking. The importance of HCM has been known for a century, but it remains a poorly understood phenomenon.

The histopathological postmortem appearance of HCM is highly variable. The predominant classical pattern is sequestration of infected red blood cells (iRBC) and hemorrhages, but iRBC cytoadherence is highly variable so that parasite sequestration may occur without any obvious vascular pathology [6]. iRBC sequestration and pathological alterations may be lacking despite clinical HCM diagnosis, perhaps due to anti-malarial treatment [5], [7]–[9]. Further, iRBC sequestration has been observed in the absence of HCM symptoms [10], [11]. Despite this histopathological variability, many consider sequestration of *P. falciparum* iRBC necessary for HCM development [12], [13]. However, HCM pathogenesis may be more complex, because different pathophysiological features are associated with HCM in African children vs. non-immune Thai adults [9] and death can occur from severe complications of *P. vivax* infections despite minimal sequestration of these parasites in the brain [10]. For these reasons, it has long been suspected that multiple mechanisms, including mechanical vascular obstruction and humoral immune responses, contribute to a fatal outcome of this severe neurological complication [14].

A widely used and well-characterized model for experimental cerebral malaria (ECM), CBA/CaJ, SW, or C57Bl/6 mice infected with *P. berghei* ANKA (PbA), shares certain similarities with *P. falciparum* HCM [15]. As for HCM, PbA-infected mice develop neurological signs including hemi- and paraplegia, ataxia, and convulsions [16]–[21]. Inflammatory cytokines are upregulated in both ECM and HCM brains, and reduced blood flow and increased lactate production are found in the brains of both mice and humans [15], [22]. *P. falciparum* and PbA induce endothelial activation with expression of adhesion molecules such as ICAM-1, VCAM-1, P- and E-selectin, which are low in the normal brain, but upregulated in infected humans and mice [23]. Finally, ECM and HCM are characterized by severe vasculopathy, i.e. platelet activation and accumulation within the cerebral microvasculature, coagulopathy, vascular leakage, edema, and microhemorrhages.

However, PbA iRBC lack knobs and specific sequestration ligands expressed on *P. falciparum* iRBC [24]. PbA iRBC can accumulate in the murine brain under certain circumstances, but whether iRBC arrest is required for ECM development and whether the underlying mechanism qualifies as true sequestration has been a matter of debate [13], [15], [23], [25]–[27]. For example, postmortem analyses of PbA-infected mice demonstrated cerebral and cerebellar capillaries packed with iRBC, which was interpreted by some as iRBC sequestration being the proximal cause of ECM [27]–[32]. Another argument in favor of iRBC sequestration is based on the finding that PbA iRBC accumulation was observed in brain sections from young mice that developed ECM, but not from older mice that had recovered from their neurological signs and later succumbed to severe anemia [32]. However, yet other studies demonstrated little or no iRBC accumulation in the murine brain and also failed to demonstrate a correlation between iRBC sequestration and ECM [13], [19], [33]. Because of the discrepancy in parasite sequestration, the value of the PbA model, in particular its relevance for screening of therapeutic interventions of HCM, has been questioned [13]. Nevertheless, PbA-infected mice do exhibit substantial platelet sequestration and leukocyte marginalization (Movila, Nacer, and Frevert, manuscript in preparation), processes that can contribute to severe malaria in children [34], so that many consider this model suitable for study of certain aspects of the pathogenesis of pediatric HCM [5], [23], [34]–[38]. In fact, it has been argued that if microvascular obstruction were to cause HCM, adhering leukocytes could replace sequestering iRBC as the major obstructive agent in ECM [39]. Some of the conflicting findings associated with the histopathology in the PbA model may be explained by the lack of a universal protocol for ECM analysis [23], [28]–[30], [32], [33], [40]–[51].

Monitoring luciferase-expressing parasites in live mice has overcome some of these technical challenges and demonstrated the requirement for simultaneous brain accumulation of PbA iRBC and CD8+ effector T cells [19], [52]–[55]. However, whole mouse imaging cannot distinguish between true iRBC adhesion to the vascular endothelium and mechanical trapping in microvessels occluded by mechanisms associated with inflammation or coagulation. Intravital microscopy (IVM) has been used to characterize the interaction between various protozoan parasites including *Plasmodium, Toxoplasma,* and *Leishmania* and internal organs of their respective hosts [56]–[61], to determine the time of *Trypanosoma brucei* spp. entry into the brain [62], and to study the cortical blood flow in various experimental models [56], [63]–[67].

*Plasmodium* iRBC, platelets, and immune cells have been suggested to accumulate in the central nervous system during ECM [65], [68]. To explore the dynamics of parasite accumulation in the brain of mice with signs of cerebral malaria, we monitored iRBC in the cortical microvasculature by intravital confocal imaging in combination with various fluorescent markers. Dynamic imaging in conjunction with a systematic comparison of the effect of PbA, *P. berghei* NK65 (PbNK), PyXL, and *P. yoelii* YM (PyYM) on the histopathology of the brain, integrity of the BBB, and manifestation of neurological signs in various susceptible and resistant mouse strains revealed that PbA iRBC transiently come into close contact with capillary endothelia. The observation that widespread vascular leakage occurs from postcapillary venules (PCV), i.e. downstream from the capillary bed, is significant, because the cerebral microvascular system contains two functionally distinct blood brain barriers (BBB), a physiological and a neuroimmunological BBB, which are formed by capillaries (diameter ≤6 µm) and postcapillary venules (diameter 10–60 µm), respectively [69]. While the physiological BBB consists of a single layer composed of endothelia, gliovascular membrane, and astrocyte endfeet, the neuroimmunological BBB encompasses two layers, the vascular endothelia with their basement membrane and the glia limitans with associated basement membranes and astrocyte endfeet, which are separated by the perivascular (Virchow-Robin) space [69]. Under inflammatory conditions, immune cells enter the CNS at the neuroimmunological BBB by first migrating across the vascular wall into the perivascular space (PVS) and then crossing the glia limitans into the parenchyma. We present, to our knowledge, the first direct *in vivo* demonstration of a difference in barrier function between the physiological and the neuroimmunological BBB in a parasitic disease.

## Results

### Parasitemia and clinical signs

To obtain a better understanding of the pathogenesis of ECM, we monitored parasitemia, clinical signs, histopathology, and BBB integrity in mice infected with PbA, PbNK, PyXL, or PyYM. Quantitative assessment of ECM-associated neurological signs was performed using the Rapid Murine Coma and Behavior Scale (RMCBS) with values of 3–7 defined here as severe, 8–17 as mild, and 18–20 as no ECM [70]. In variation of previous studies, we infected mice at the age of 3 weeks. Because young children are predominantly susceptible to neurological complications during *P. falciparum* infections, we tried to approximate a similar level of immune maturity in our animal model. Since the immunological maturity of mice at 1 week is considered similar to newborn humans [71] and at 6 weeks mice have mature immune systems, we infected mice at 3 weeks of age. The equivalence of immune maturity in young children and 3-week-old mice is not validated, but we consider this variation from previous studies to be reasonable.

To characterize the factors that contribute to the development of ECM vs. hyperparasitemia (HP) in ECM susceptible vs. resistant hosts, we selected four mouse strains for a systematic comparison under standardized infection conditions: SW and CBA/CaJ mice which were previously described as susceptible to PbA induced ECM, Balb/c mice which are known to be resistant to PbA-induced ECM, and A/J mice which were originally reported to respond to PyXL infection with a cerebral syndrome similar to HCM [72].

**Figure 1:**
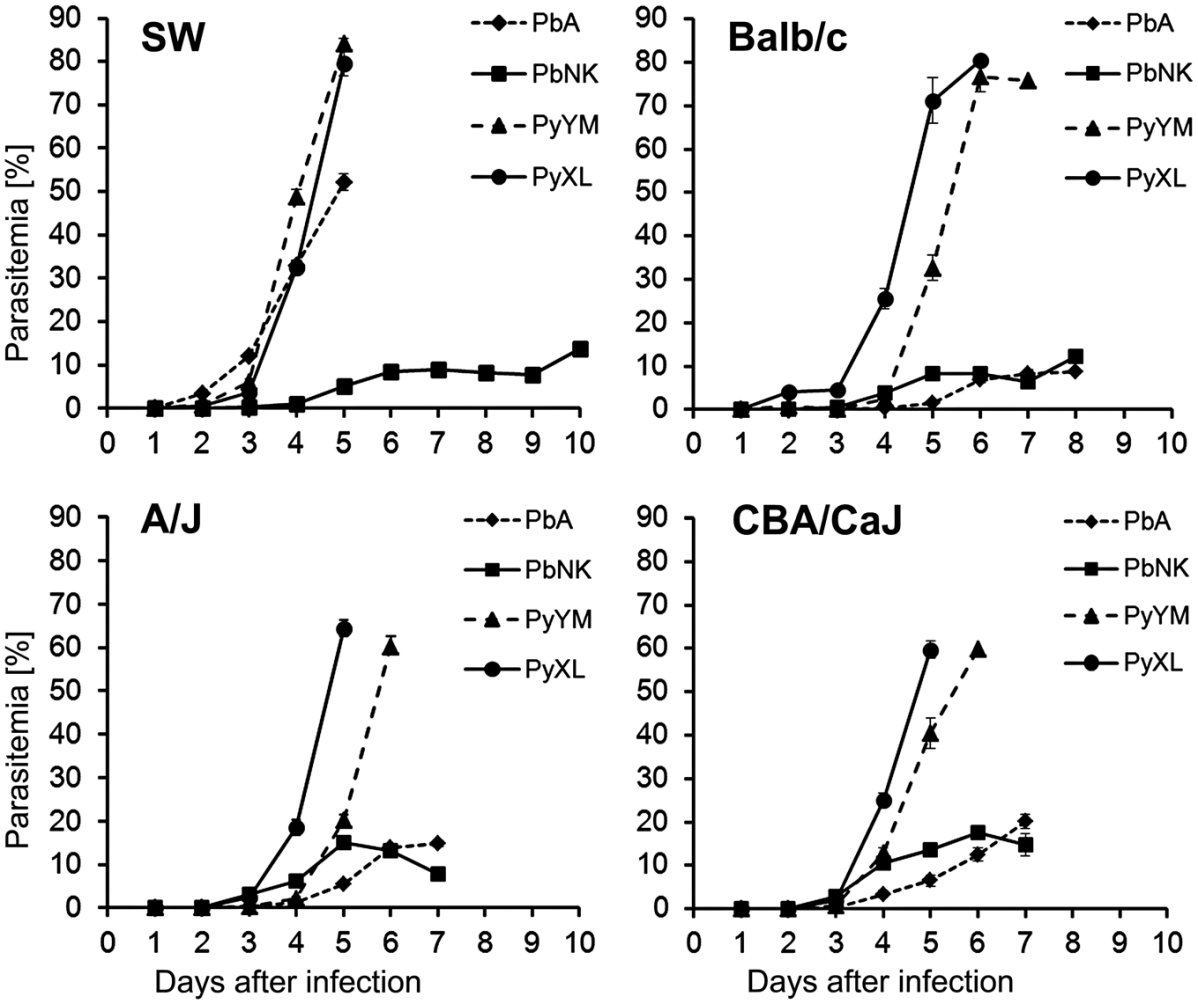
Time course of parasitemia in young mice of different genetic background. Groups of 6 three week-old SW, Balb/c, A/J, or CBA/CaJ mice were infected with 10^6^ iRBC of the indicated parasite lines. As expected, infection with PbA resulted in a slow rise in parasitemia, which typically reached a plateau at below 20%. While PbA-infected SW mice developed ECM signs at around 50% parasitemia on day 5, CBA/CaJ mice displayed ECM signs on day 6 after infection. Balb/c and A/J were resistant. PbNK-infected CBA/CaJ mice also developed ECM. In contrast, infection with PyXL and PyYM resulted in a rapid rise in parasitemia between days 4 and 5 irrespective of the mouse strain. Mice were typically moribund by day 5 with >60% parasitemia. Data represented the mean parasitemia ± SD.

Three week-old CBA/CaJ and SW mice responded to PbA infection with neurological signs and died from ECM just like adult mice used by others for similar studies in the past [5], [23], [28]–[30], [32]–[38], [40]–[51], while young A/J and Balb/c mice were resistant (**Fig. 1** **and** **Table 1**). The parasitemia in PbA-infected CBA/CaJ mice typically remained below 20%, whereas it reached 40–60% in SW mice at terminal stages of the disease (**Fig. 1**) [73]. Unexpectedly, 50% of the PbNK infected CBA/CaJ mice also responded with BBB disruption and neurological impairment, while SW, Balb/c, and A/J mice were resistant (**Table 1**). PbA or PbNK infected mice with symptomatic ECM presented with ataxia, limb paralysis, a poor righting reflex, seizures and clonic convulsions with peddling of the legs, respiratory distress, rollover, and coma between days 6–8 after infection. Neurological signs first became apparent at parasitemia levels around 7% on day 5 or 6 after infection. In agreement with the generally low parasitemia, ECM mice exhibited a normal skin color and excreted clear urine. In contrast, PyXL and PyYM induced a rapid rise in parasitemia in all mouse strains leading to HP (>60%) and death within 5–7 days after infection (**Fig. 1**). In agreement with the extensive haemolysis associated with HP, PyXL and PyYM infected mice presented with paleness, secretion of biliverdin into the urine, rough and dull coat, hunched posture, and lethargy. Neurological signs were not observed. Thus, lethal strains of *P. berghei* and *P. yoelii* kill susceptible young mice via ECM and HP, respectively, two different manifestations of severe malaria.

**Table 1:** Neurological impairment and integrity of the BBB.

Parasite strain	Mouse species	ECM-positive/Total	OD620 (± SEM)
**PbA**	**SW**	6/9^†^	0.224 (0.012)***
**PbNK**		0/3	0.082 (0.003)
**PyXL**		0/6	0.080 (0.004)
**PyYM**		0/6	0.085 (0.008)
**Uninfected**		0/3	0.053 (0.006)
**PbA**	**Balb/c**	0/3	0.042 (0.002)
**PbNK**		0/3	0.032 (0.002)
**PyXL**		0/3	0.047 (0.004)
**PyYM**		0/3	0.069 (0.009)
**Uninfected**		0/3	0.045 (0.003)
**PbA**	**A/J**	0/3§	0.173 (0.056)
**PbNK**		0/3	0.109 (0.005)
**PyXL**		0/3	0.079 (0.011)
**PyYM**		0/3	0.053 (0.006)
**Uninfected**		0/3	0.042 (0.004)
**PbA**	**CBA/CaJ**	2/6	0.404 (0.100)**
**PbNK**		3/6	0.184 (0.033)*
**PyXL**		0/3	0.054 (0.004)
**PyYM**		0/3	0.036 (0.004)
**Uninfected**		0/3	0.047 (0.003)

Groups of 3 *Plasmodium*-infected mice, unless otherwise indicated, were injected with Evans blue at first sign of ECM (typically on day 6 after infection) or at a parasitemia exceeding 50% (typically on day 5), and sacrificed three hours later by exsanguination under anesthesia. Brains were extracted with formamide and vascular leakage was determined by measuring Evans blue absorption at 620 nm (OD620).

*** *P*<0.001,

** *P*<0.01,

* *P*<0.05 indicate a significant increase in BBB permeability. Data were analyzed separately within each mouse strain by one-way ANOVA followed by Dunnett's test for comparisons with a control.

§ Two PbA-infected A/J mice displayed elevated OD620 values, but no neurological signs.

### Histopathology of the brain

Next, we assessed the histopathological alterations in the brains of infected SW, CBA/CaJ, A/J, and Balb/c mice in response to ECM vs. HP. Mice selected for histological analysis exhibited clear neurological signs and ranged at 3–7 of the RMCBS. All mice were exsanguinated prior to brain harvesting to avoid misinterpretations based on agonal cerebral congestion.

**Figure 2:**
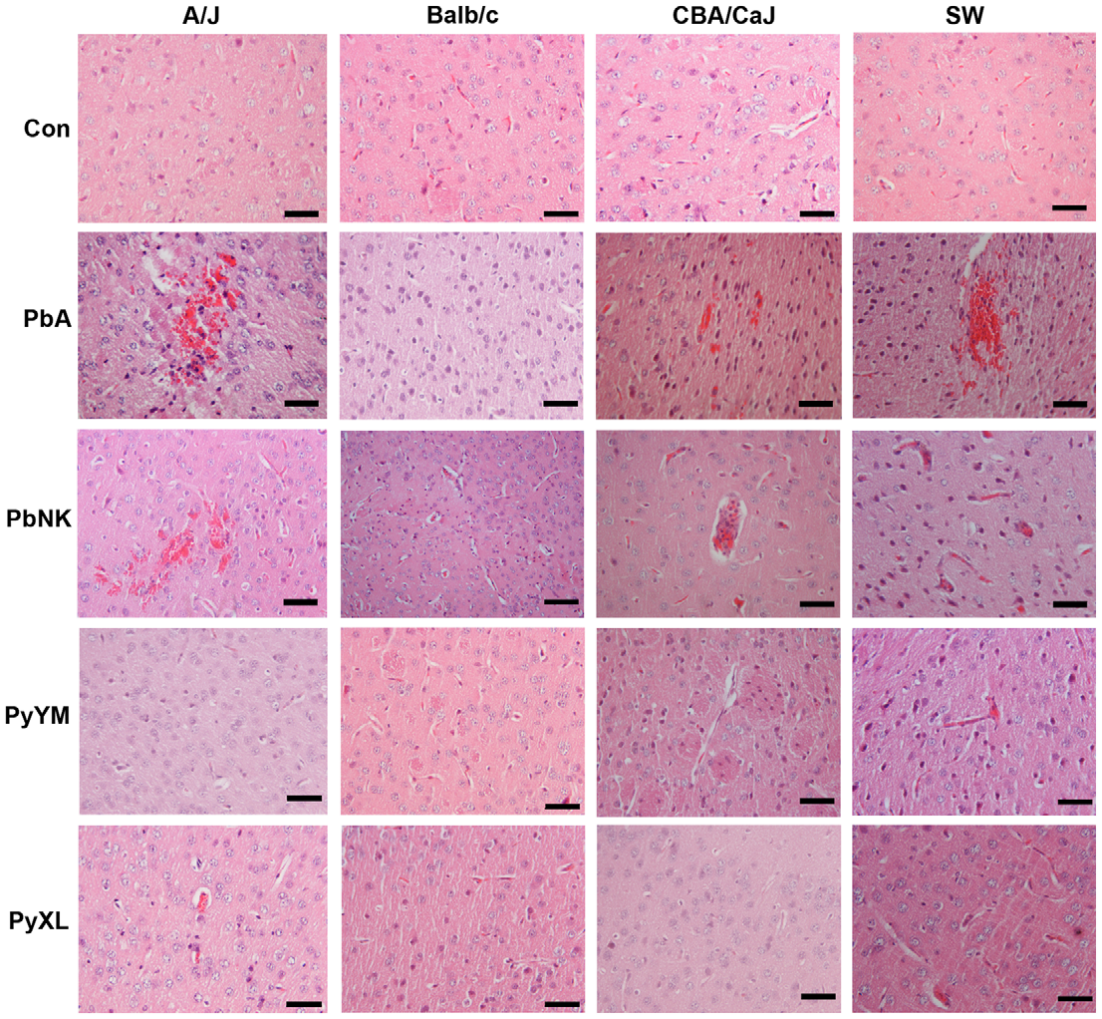
Histopathology of *P. berghei* and *P. yoelii* infection in different mouse strains. Representative images of brains from groups of 3 A/J, Balb/c, CBA/CaJ, or SW mice, infected with PbA, PbNK, PyXL, or PyYM, as well as uninfected control brains were paraffin embedded and sections stained with H&E. PbA and PbNK infection caused focal vascular congestion and hemorrhages throughout the gray and white matter in the brains from A/J, CBA/CaJ, and SW, but not Balb/c mice. Brains from PbA-infected mice with symptomatic ECM exhibited congested microvessels containing leukocytes, which were restricted to the vascular lumen. Other than rare foci of large numbers of iRBC, the microvasculature of PyXL- or PyYM-infected brains exhibited no histological alterations in any of the mouse strains compared to uninfected controls. All images derive from coronal brain sections at Bregma and correspond to neocortical layer I tissue. Scale bars = 50 µm.

As expected, coronal H&E stained paraffin brain sections from symptomatic PbA-infected CBA/CaJ and SW mice revealed the classical hallmarks of ECM such as petechiae and hemorrhages within the gray and white matter, and occasional intravascular leukocytes, which did not enter the perivascular Virchow-Robin space or the parenchyma unless associated with hemorrhages (**Fig. 2**). In contrast to earlier reports using this model [32], [72], there was no evidence for microvascular occlusion caused by large numbers of sequestered iRBC, leukocytes, intravascular macrophages with phagocytozed hemozoin, or dilation of the Virchow-Robin spaces indicating cerebral edema. Interestingly, some of the PbA and PbNK infected A/J mice, while asymptomatic, presented histopathological alterations typical for ECM (**Fig. 2**). No pathological changes were found in brains from PbA-infected Balb/c mice. The PbNK infected CBA/CaJ mice with neurological signs (3/6; Table 1) also exhibited histopathological changes typical for ECM, while brains from SW and Balb/c mice appeared normal, as anticipated (**Fig. 2**). None of the brains from PyXL or PyYM infected mice with HP exhibited evidence for hemorrhages, edema, or inflammatory infiltrates (**Fig. 2**). In contrast to Yoeli's original report [72], congested (“ballooning”) cerebral microvessels that contained large numbers of iRBC were rarely detected in a total of 9 mice examined.

### IVM of the cortical microvasculature

Because histopathological analysis cannot distinguish between cells traveling at bloodstream velocity from those rolling along the vascular wall, being firmly attached and stationary, or forming aggregates that obstruct the blood flow, we established an intravital brain imaging procedure suitable for mice suffering from late-stage ECM or HP using SW mice infected with PbA-GFP or PyXL-RFP. Confocal IVM was performed through a cover-slipped cranial window, which allows examination of a considerably larger area (∼20,000 µm^2^) compared to the thinned skull technique (∼30 µm^2^), which also requires two-photon microscopy for penetration of the remaining layer of bone [74], [75]. The Dura mater was preserved throughout this study to prevent injury to the cerebral cortex, however the pial vasculature was excluded from study. IVM analyses were performed exclusively on microvessels located within the neocortical layer I of the murine brain [76], i.e. beyond the 10–15 µm thick meninges (see also Materials & Methods). IVM of severely sick animals is technically challenging, but optimization of protocols for anesthesia, craniotomy, and IV injection of fluorescent markers allowed us to obtain good recordings of approximately 70% of the ECM mice and 50% of the HP mice. The total number of successfully imaged mice included in this study was: 23 PbA-infected mice at the time of ECM (typically between days 6 and 7), 1 PbA-infected mouse before ECM (day 5), 2 PbA-infected mice that failed to develop ECM (day 8), 10 PbA-infected and FTY720 or imatinib treated mice that survived the critical period of ECM development (day 8), 20 PyXL- and 4 PyYM-infected mice with >50% parasitemia (days 5–6), and 29 uninfected control mice. Analysis of these animals allowed us to distinguish between ECM-associated vascular alterations and surgery-related artifacts. Mice with damage to the pial vasculature exhibited extensive bleeding and profuse leakage of the vascular marker. Such animals did not produce useful data and were excluded from study.

### iRBC behavior in the cortical microvasculature

At the onset of severe ECM symptoms (RMCBS = 3–7, PbA-GFP infection) or at >50% parasitemia (PyXL-RFP infection), mice were anesthetized, subjected to craniotomy, and immobilized on the stage of an inverted microscope. Placement of the cranial window over the parietal cortex allowed imaging of cortical branches of the anterior cerebral artery, smaller arterioles down to capillary size, PCV, and superficial parietal veins that drain into the superior sagittal sinus. To visualize the lumen of the microvasculature, mice were injected prior to surgery with fluorescent BSA or Evans blue. Uninfected RBC as well as all other non-fluorescent blood cells exclude the vascular marker and appear as negatively stained dark ovals or streaks of velocity-dependent diameter and length (**Fig. S1, Video S1 and S2**).

**Figure 3:**
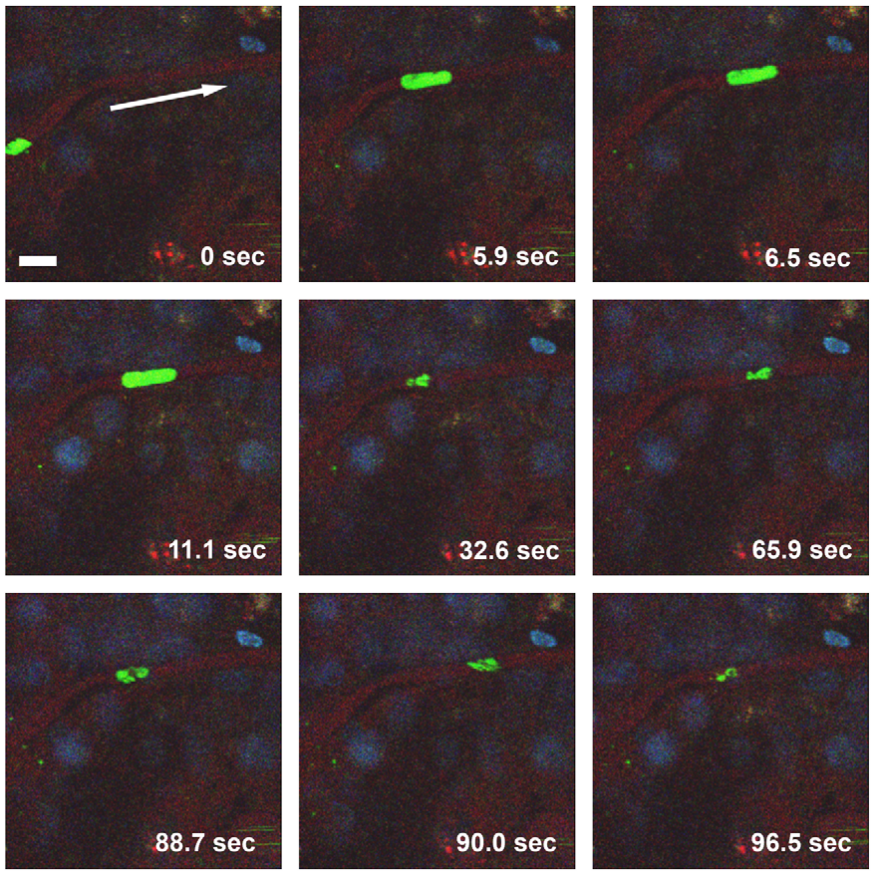
iRBC make intimate contact with capillary endothelia. Selected frames from a two-minute IVM movie, which shows several late- and early-stage PbA-GFP iRBC traveling through a capillary. While iRBC harboring early-stage parasites (green, small) travel at blood velocity, iRBC containing late-stage parasites (green, large) squeeze slowly through the narrow capillary lumen, presumably due to the known increase in iRBC rigidity. Note the elongated shape of the two iRBC that contain large parasites, most likely trophozoites or schizonts. The vascular lumen is labeled with BSA-TX (red); nuclei are stained with Hoechst (blue). The arrow in the top left (0 sec) panel indicates the direction of blood flow. Scale bar = 10 µm. **Video S3.**

**Figure 4:**
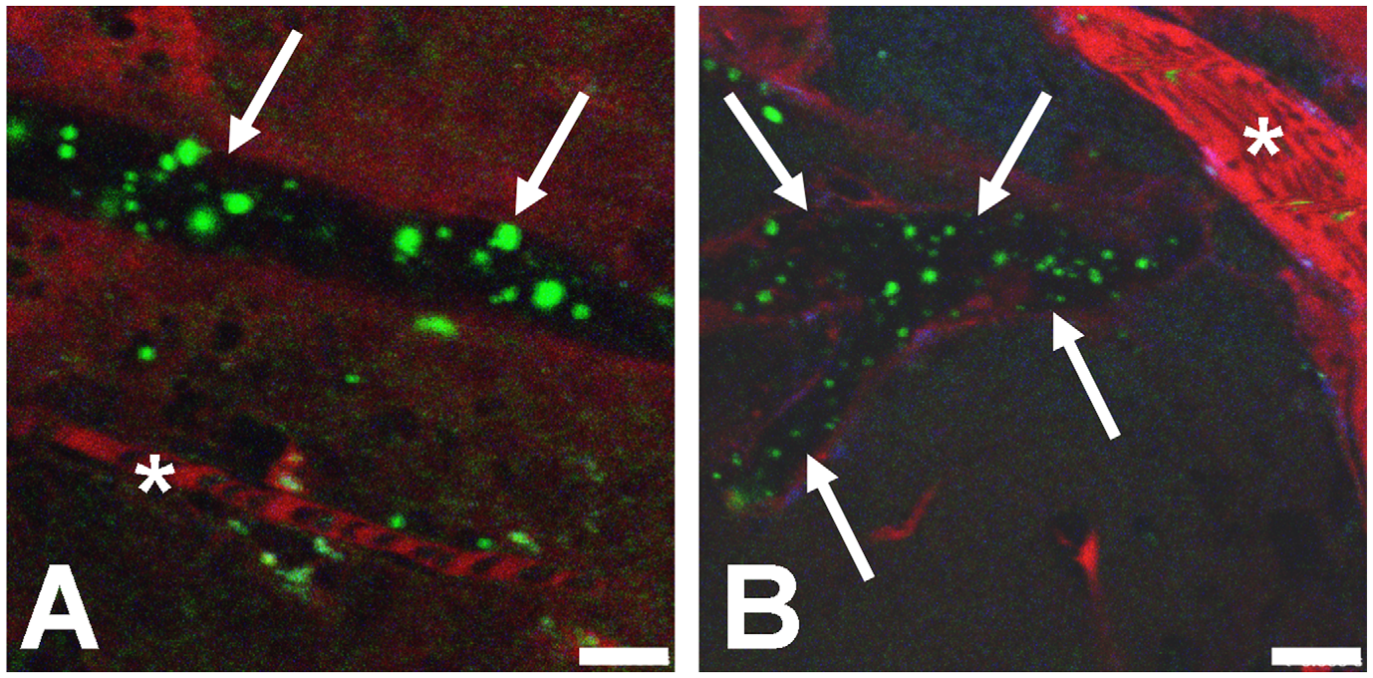
Microvascular occlusion can cause iRBC trapping. A) A blocked larger vessel contains PbA-GFP iRBC (arrows), while a neighboring capillary (white star) displays normal blood flow. B) A large number of iRBC are visible in a blocked vessel (arrows) while a neighboring vessel (white star) exhibits normal flow. The vascular lumen was labeled with BSA-TX. Scale bars = 10 µm. **Video S4 and S5.**

To explore the possibility that PbA iRBC adhere to the microvascular endothelium, we monitored individual microvessels for iRBC arrest and blockage of the blood flow. In SW mice, iRBC containing small early-stage parasites generally passed through capillaries without any apparent reduction in speed. In contrast, iRBC that harbored large late-stage parasites, trophozoites or schizonts, decelerated by more than 3 orders of magnitude, from the normal RBC velocity in brain capillaries of 2±1.4 mm/s [77] to 1.2±0.83 µm/s (N = 3) (**Fig. 3** **, Video S3**). Due to their large size, these mature iRBC became distorted while squeezing through the narrow capillary lumen, possibly leading to endothelial dysregulation due to temporary hypoperfusion, similar to the pathology associated with acute trauma in humans. In larger vessels such as arterioles or PCVs, iRBC travelled at bloodstream velocity and cytoadhesion was not observed. A similar reduction in PbA iRBC velocity was not observed in CBA or Balb/c mice, although mature parasite stages were present in the blood circulation (data not shown). While the blood flow of the microvasculature was generally unimpaired, some microvessels of different diameters were occluded as indicated by focal absence of the vascular marker (**Fig. 4** **, Video S4 and S5**).

Most iRBC that harbored early-stage PyXL-RFP parasites traveled at bloodstream velocity. Some late-stage iRBC passed through capillaries at a somewhat slower speed in SW mice (**Fig. S2, Video S6**), but generally traveled at bloodstream velocity in PyXL- or PyXNL-infected CBA mice. Although “ballooning” vessels such as those described by Yoeli and Hargreaves [72] were rarely observed, capillaries and larger microvessels were occasionally occluded. The iRBC high density in these vessels was due to the parasitemia exceeding 50% at the time of imaging. The fluorescent plasma marker was excluded from blocked vessels suggesting that interruption of the blood flow occurred prior to surgery and intravital imaging. Neighboring vessels, often of a smaller size (**Fig. S3, Video S7**), remained perfused also indicating that vascular obstruction was caused by the infection, not experimental procedures.

Thus, while some iRBC, in particular PbA-infected reticulocytes harboring large late-stage parasites, make intimate contact with the vascular endothelium while squeezing slowly through the narrow capillary lumen, iRBC generally travel at bloodstream velocity and do not arrest.

### BBB integrity

**Figure 5:**
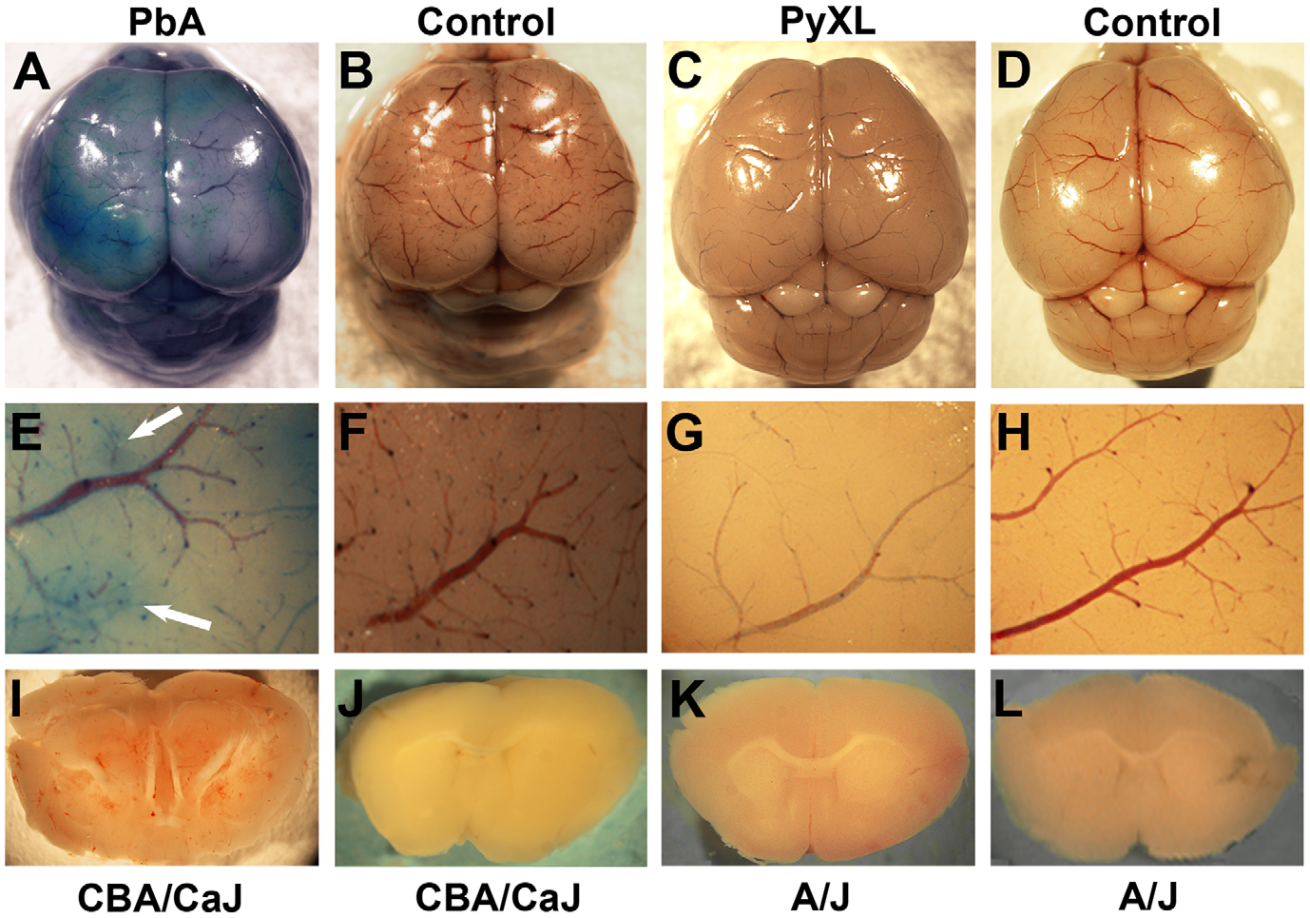
Blood brain barrier integrity during ECM and HP. A–D) Appearance of the cortical microvasculature of CBA/CaJ mice infected with PbA (A) no parasites (B) and A/J mice infected with PyXL (C) or no parasites (D). Mice were injected with Evans blue upon appearance of neurological signs (A) or reaching >50% parasitemia (B). Note that focal leakage of the vascular tracer into the parenchyma occurs during ECM (A), but not HP (C), and is absent from uninfected control mice. E–H) Higher magnification of the cortical microvasculature of mice infected with PbA (E), PyXL (G), or no parasites (F and H). Note the petechiae and diffuse Evans blue staining of the PbA-infected brain (E, arrows). I–L) Coronal slices of brains infected with PbA (I), PyXL (K), or no parasites (J and L). Numerous small hemorrhages are visible in both the gray and the white matter of the PbA-infected (I), but not the PyXL (K) or uninfected brains (J, L). The figure shows representative images of 3 mice per group.

Next, we determined whether neurological signs were associated with BBB dysfunction. We chose Evans blue as a vascular marker because of its ability to bind to albumin [78], [79], reasoning that plasma protein leakage across the BBB into the cerebral parenchyma reflects the pathologically relevant mode of vascular injury. Only brains from PbA or PbNK infected mice with symptomatic ECM appeared macroscopically blue, while those from PbA or PbNK infected mice without ECM, from all PyXL and PyYM infected mice, and from all control mice retained their normal pink color (**Fig. 5**). Brains from mice with severe neurological signs appeared visually darker blue than those from mice with mild ECM (data not shown), suggesting a close correlation between neurological involvement and BBB disruption.

To quantify vascular leakage, we measured the absorption of Evans blue at 620 nm (OD620) in formamide extracts (**Table 1**). Evans blue was administered upon development of severe ECM (RMCBS = 3–7) or HP (RMCBS = 20), 6–7 or 4–5 days post-infection, respectively. As expected, brains from PbA-infected SW and CBA/CaJ mice with symptomatic and histopathologically confirmed ECM exhibited a significant OD620 elevation (ANOVA SW: F_(4)_ = 12.54; *P*<0.001; ANOVA CBA/CaJ: F_(4)_ = 16.96; *P*<0.001). Consistent with the relatively moderate histopathological alterations, PbA-infected A/J mouse brains displayed a non-significant OD620 increase (ANOVA F_(4)_ = 3.23; *P* = 0.06), while Balb/c mice were resistant. In contrast to other reports [31], [52], [80]–[82], but in agreement with our observation of ECM signs, PbNK infected CBA/CaJ mice exhibited significant vascular leakage (Dunnett's Test PbNK-CBA/CaJ: T_(4)_ = −4.06; *P* = 0.01), while the BBB integrity of PbNK infected (ECM-negative) SW, A/J, and Balb/c mice was preserved as expected. None of the PyXL or PyYM infected mice exhibited any significant opening of the BBB as determined by Evans blue OD620. Although this result was expected based on the lack of neurological signs, it is nevertheless remarkable in view of the extreme parasitemia and haemolysis caused by these lethal *P. yoelii* strains. Thus, BBB disruption is correlated with symptomatic ECM, but not HP.

### Time course of BBB dysfunction

Microscopic inspection of the cerebral cortex from PbA-infected CBA/CaJ mice with severe ECM on day 6 (RMCBS = 3–7) revealed small petechiae and focal diffusion of Evans blue into the surrounding tissue (**Fig. 5A and 5E**). Similar results were obtained in CBA/CaJ mice infected with PbNK (data not shown). Unlike HCM, where hemorrhages are largely restricted to white matter, coronal slices of these ECM brains exhibited microhemorrhages both in the gray and white matter (**Fig. 5I**). Other than evidence for severe anemia, A/J mice infected with PyXL (**Fig. 5C, 5G, and 5K**) or PyYM (not shown) did not exhibit vascular alterations. No alterations were found the corresponding uninfected CBA/CaJ (**Fig. 5B, 5F and 5J**) or A/J control mice (**Fig. 5D, 5H, and 5L**).

To obtain insight into the early events of BBB disruption, we injected groups of two CBA/CaJ mice with Evans blue 4, 5 or 6 days after infection with PbA and analyzed the cortical surface and coronal brain sections at microscopic resolution. Compared to the uninfected control brain (**Fig. S4A**), infected brains appeared unusually pale on day 4, i.e. well before the onset of neurological signs (RMCBS = 18–20) (**Fig. S4B**). Subsequently, brains continued to appear ischemic and the color intensified after PbA infection from a faint bluish shade on day 5 (RMCBS = 20) (**Fig. S4C**) to dark blue on day 6 at the time of severe neurological signs (RMCBS = 3–7) (**Fig. S4D**). As mentioned above, the intensity of EB leakage was correlated with the severity of neurological signs.

### Site of BBB dysfunction in live mice

**Figure 6:**
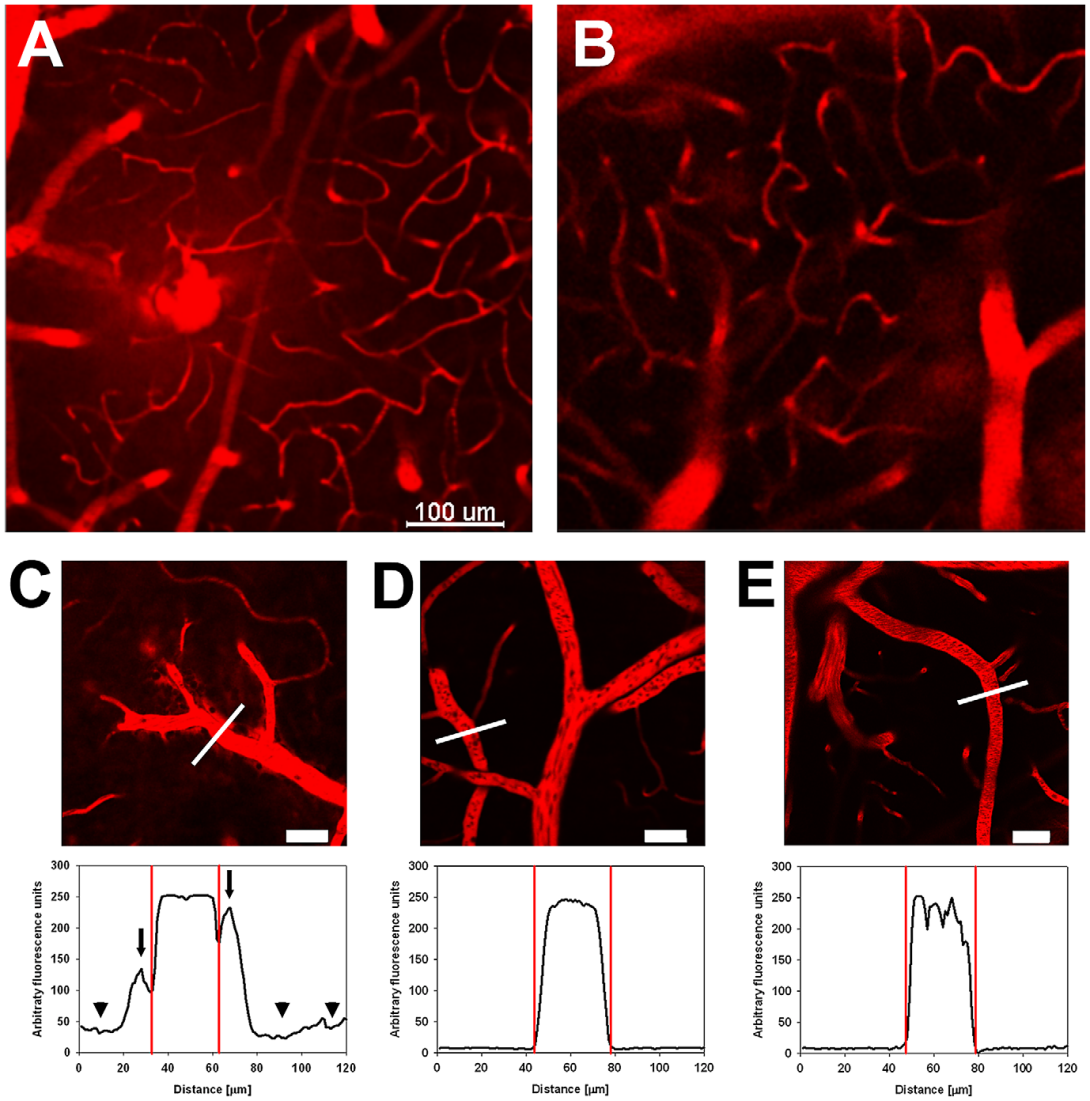
Site of the ECM-associated BBB opening within the cerebral vascular tree. CBA/CaJ mice were infected with PbA, PyXL, or no parasites. Microhemorrhages were occasionally found in the cortical vasculature of PbA-infected mice with ECM (A), but not PyXL-infected mice with HP (B). C–E, upper panels: C) PbA-infected mice exhibited extensive Evans blue leakage from PCV, but not capillaries, into the PVS and the parenchyma. In contrast, there was no Evans blue leakage into the cerebral parenchyma of PyXL (D) or age-matched uninfected control mice (E). C–E, lower panels: Graphic representation of Evans blue emission in relation to PCV from PbA, PyXL, and control mouse brains. The graphs show the Evans blue emission profile along the lines shown in (C–E). Note the emission peaks on either side of the PCV in the PbA-infected brain, which represent Evans blue leakage into the PVS (arrows). These peaks are absent in the PyXL-infected and control brain. Further, the overall Evans blue signal in the parenchyma surrounding the PCV is elevated in the PbA-infected brain (C, arrowheads) compared to the controls (D, E). The serrated shape of the control profile (E) is due to the presence of (dark) intravascular RBC. Scale bars = 100 µm (A, B), 50 µm (C–E).

**Figure 7:**
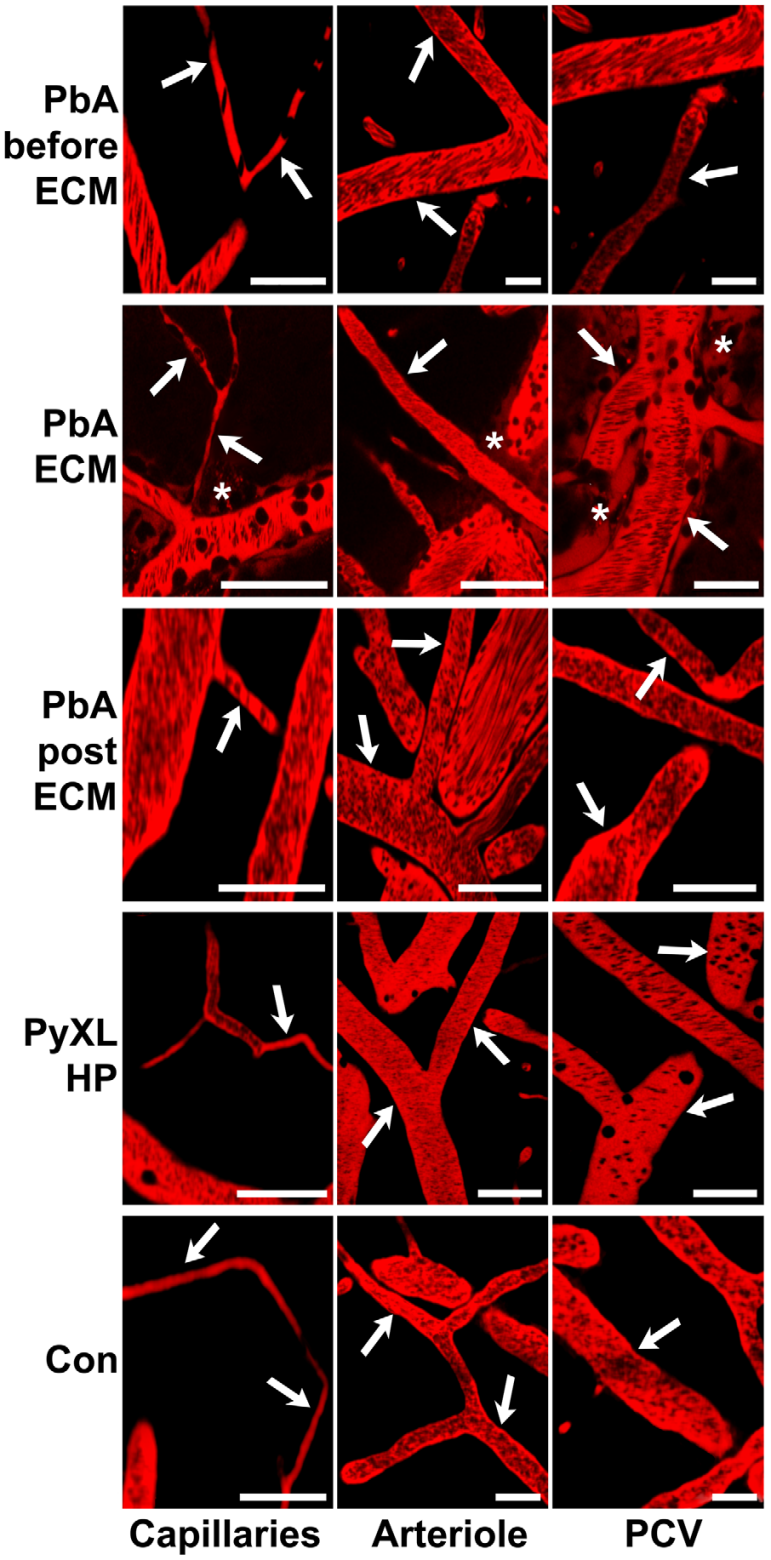
Site of neuroimmunological BBB opening in symptomatic ECM mice. Capillaries, arterioles, and PCV from PbA-infected CBA/CaJ mice before ECM (day 5), at the time of neurological signs (typically between days 6 and 7), and post ECM (day 8). The images show that leakage of Evans blue occurs only from PCV and only during, not before or after, symptomatic ECM. Neither PCV nor arterioles or capillaries from PyXL-infected mice with HP or uninfected control mice (Con) exhibit any leakage. Scale bars = 50 µm.

Next, we used IVM of CBA/CaJ mice to determine the site of Evans blue leakage into the parenchyma of the brain. Leakage of intravenously injected Evans blue revealed occasional microhemorrhages in PbA-infected mice, but not in PyXL-infected mice with HP or in uninfected control mice (**Fig. 6A and 6B**). However, the relatively rare occurrence and highly focal nature of these vascular injuries was difficult to reconcile with the homogenous Evans blue staining of ECM brains (**Fig. 5A** **and** **S4D**). Mice with ECM exhibited Evans blue leakage from larger microvessels (diameter 10–60 µm) across the perivascular space into the neuropil (**Fig. 6C**). Size and direction of blood flow identified these vessels as PCV, the morphological correlate of the neuroimmunological BBB [69]. In addition, PCV could be distinguished from similarly sized arterioles by differential CD14/CD31 labeling of the endothelium (**Fig. S5**): 1) While PCV from PbA-infected mice with ECM expressed CD14, PyXL-infected mice with HP or uninfected control mice were CD14-negative. Arterioles from all infected and uninfected mice were consistently CD14-negative. 2) While PCV constitutively exhibited a low level of CD31 expression that did not change in response to infection, arterioles were consistently CD31-positive. Neither cortical arterioles (diameter 10–60 µm), which are also surrounded by a Virchow-Robin space, nor capillaries (diameter ≤6 µm) exhibited any detectable leakage under the same standardized imaging conditions (**Fig. 7**) suggesting that the physiological BBB [69] remained intact in mice with ECM. Further, no leakage from PCV was detectable on day 5, i.e. before the onset of ECM symptoms (RMCBS = 20), or on days 8 and 9, i.e. in mice that failed to develop neurological signs (RMCBS = 20) and progressed to high parasitemia (**Fig. 7**). Because IVM is restricted to examination of the superficial cortical microvasculature, we injected CBA/CaJ mice with clear neurological signs (RMCBS = 3–7) and uninfected control mice with Evans blue and prepared coronal vibratome sections of live brain tissue. Bright-field macrographs and confocal IVM demonstrate that Evans blue leakage occurs throughout the parenchyma of the cerebrum (**Fig. S6 and Video S8**) and cerebellum (data not shown) thus confirming our dynamic *in vivo* results. Intravital labeling of vascular endothelia with anti-PECAM-1, necrotic cells with propidium iodide, and apoptotic cells with YOPRO-1 provided no evidence for disruption of or injury to the endothelial lining (data not shown) suggesting that BBB dysfunction involves a regulated fluid transport mechanism rather than endothelial cell death. In contrast, PyXL-infected mice with HP (**Fig. 6D**) or uninfected control mice (**Fig. 6E**) showed no evidence for vascular leakage from PCV, arterioles, or capillaries.

### Mechanism of vascular leakage

**Figure 8:**
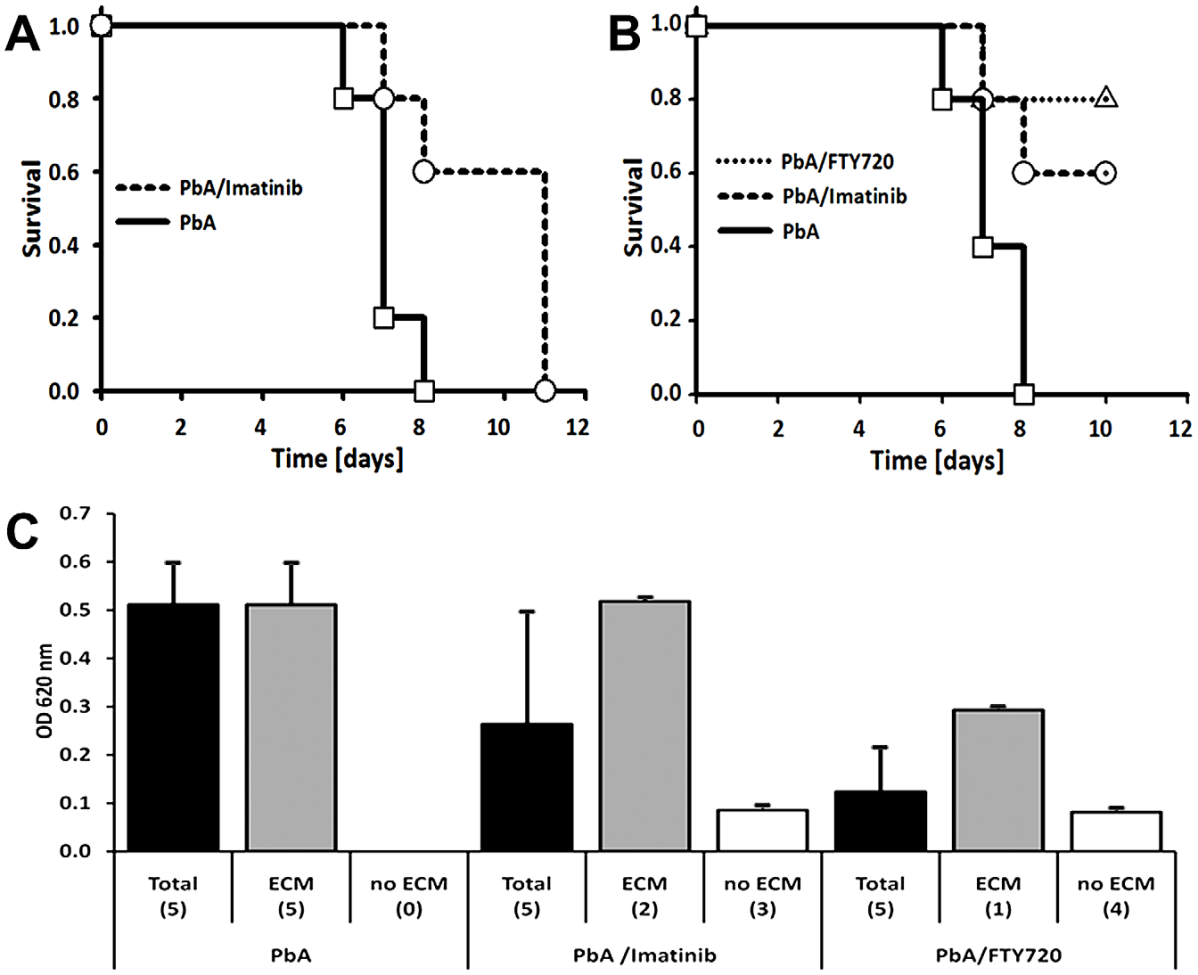
Vascular leakage is regulated and preventable. Groups of 5 PbA-infected CBA/CaJ mice received two daily oral doses of 250 mg/kg imatinib on days 5, 6, and 7 post infection, one daily oral dose of 0.3 mg/kg FTY720 starting one day before infection, or 3) no treatment. A) While all PbA-infected untreated mice died with clear neurological signs between day 6 and 8, survival of the imatinib-treated mice was significantly prolonged. B) Whereas all untreated PbA-infected mice died from ECM between day 6 and 8, imatinib and FTY720 treatment allowed 80% and 60%, respectively, of the mice to survive until day 10 at which time they were sacrificed for quantification of Evans blue in the brain. C) OD620 measurement revealed Evans blue leakage into the parenchyma of the untreated controls and the mice that succumbed to ECM despite treatment, but not the treated mice that survived until day 10. The data represent mean OD620 values ± STD. Brain hemispheres were analyzed individually.

To obtain insight into the mode of the ECM-associated BBB opening, groups of 5 CBA/CaJ mice were infected with PbA as above and treated with 1) the tyrosine kinase inhibitor imatinib, which was reported to block endothelial vesicular transcytosis in the brain [83] or 2) FTY720, a modulator of the sphingosine-1-phosphate (S1P) pathway that was shown to increase survival to ECM and decrease Evans blue leakage into the brain parenchyma of PbA-infected mice [84], or 3) vehicle. While all PbA-infected vehicle-treated mice died with clear neurological signs between day 6 and 8 (mean = 7.0 days), survival of the imatinib-treated mice was significantly prolonged (mean = 9.6; Kaplan-Meier log-rank analysis: *P*<0.05) (**Fig. 8A**). Strikingly, no neurological signs were observed in the 3/5 imatinib-treated mice that survived until day 11; instead, these mice died with signs of high parasitemia (>35%; data not shown). In a separate study, all untreated control mice died between day 6 and 8 post infection as expected, while 3/5 mice treated with imatinib (Kaplan-Meier log-rank analysis: *P*<0.05,) and, as expected [84], 4/5 mice treated with FTY720 (*P*<0.05) survived the critical period of ECM development and were sacrificed on day 10 (**Fig. 8B**). OD620 measurement (**Fig. 8C**) revealed a significant reduction of Evans blue leakage into the brain parenchyma of mice treated with imatinib (analyzed on day 7, 8, and 10) and FTY720 (analyzed on day 7 and 10) compared to the group of control mice (analyzed on day 6, 7, and 8). Separate analysis of the imatinib and FTY720 treated mice that developed ECM and died on day 7 or 8 vs. those that remained ECM-negative and survived until day 10 confirmed that vascular leakage and neurological signs are directly correlated (**Fig. 8C**). Neither imatinib nor FTY720 had any effect on the course of the parasitemia until day 8 (data not shown). Together, these findings suggest that the ECM-associated opening of the neuroimmunological BBB is governed by regulated paracellular and transcellular fluid transport pathways.

### Role of LFA-1 mediated cellular interactions in ECM-associated BBB disruption

**Figure 9:**
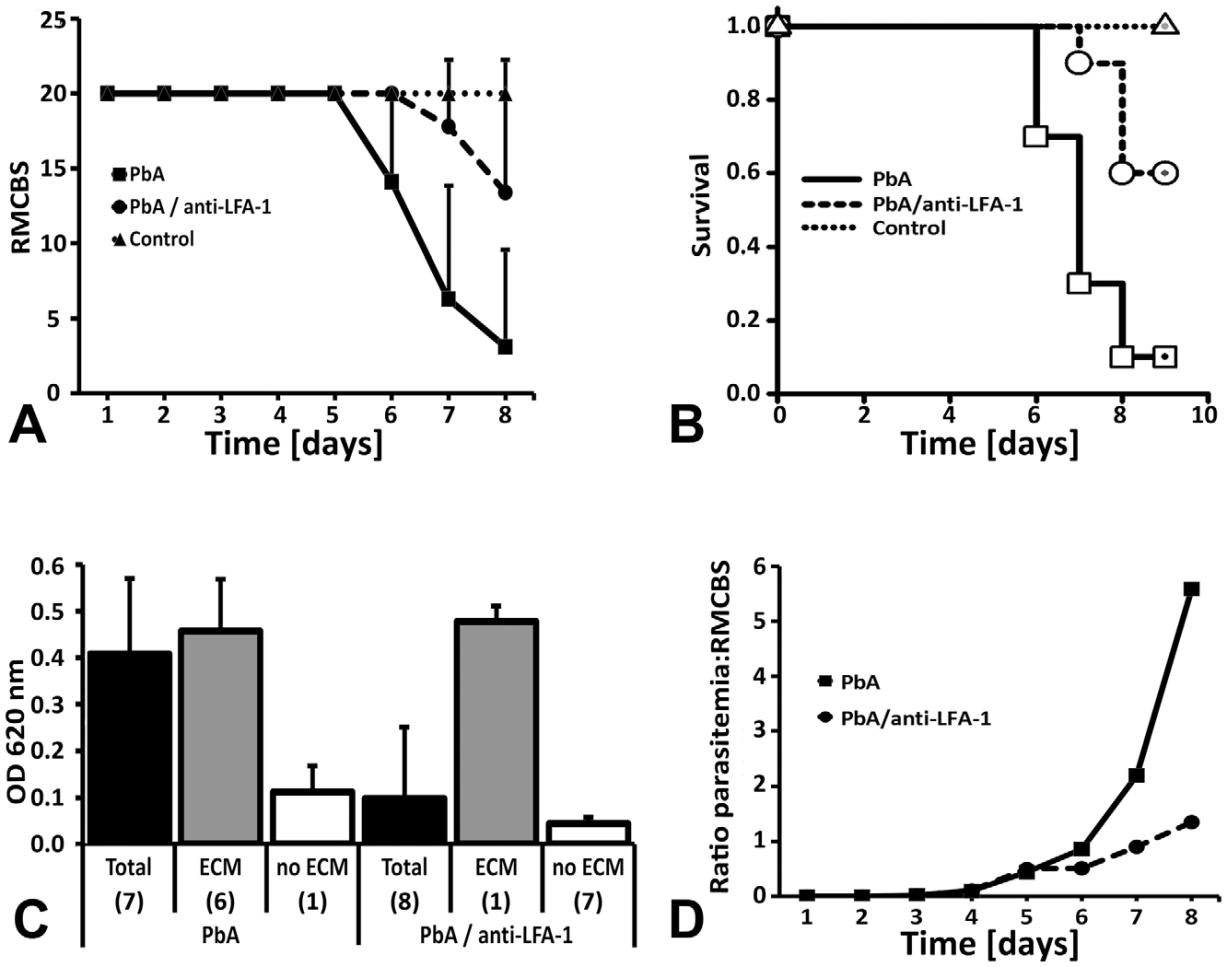
LFA-1 mediated cellular interactions contribute to BBB opening. Five days after infection with PbA, CBA/CaJ mice were inoculated with 200 µg anti-LFA-1 mAb (N = 10) or no antibody (N = 10). A) Neurological signs based on RMCBS grading were significantly reduced in anti-LFA-1 treated PbA-infected mice compared to the untreated PbA-infected control mice. The data are represented as average ± SD. B) While 90% of the PbA-infected mice (N = 10) developed severe ECM between day 6 and 8, only 40% of the anti-LFA-1 treated mice (N = 10) showed neurological signs. C) Compared to PbA-infected untreated mice (N = 7), anti-LFA-1 treated PbA-infected mice (N = 8) exhibited a significant reduction in vascular leakage in the brain. Separate graphic representation of the 1 ECM-negative and 6 ECM-positive mouse brains from the PbA group and the 1 ECM-positive and 7 ECM-negative mouse brains from the PbA/anti-LFA-1 group revealed that neurological signs and vascular leakage are directly correlated. The data represent the average OD620 measurements from the two brain hemispheres from each mouse ± SD. Three of the 10 PbA-infected mice and 2 of the 10 PbA-infected/anti-LFA-1 treated mice died overnight and were excluded from analysis. D) Depiction of the parasitemia-to-RMCBS ratio accentuates the effect of LFA-1 blockage on the development of neurological signs.

Next, we tested the hypothesis that lymphocyte function-associated antigen 1 (LFA-1, CD11a) is involved in the disruption of the neuroimmunological BBB. LFA-1 mediates the recruitment of T and B cells, macrophages, and neutrophils to sites of endothelial activation [85] and was previously reported to abrogate ECM in the PbA CBA/Ca mouse model [86], [87]. Two groups of 10 mice were infected with PbA. Five days later, i.e. prior to the expected manifestation of neurological signs, one group of mice was intravenously inoculated with mAb M17/4 against LFA-1. Anti-LFA-1 treatment had no effect on the course of the parasitemia (data not shown). While 90% of the PbA-infected mice became ECM-positive between day 6 and 8 post infection, only 40% of the anti-LFA-1 treated mice developed neurological signs; the remaining 60% survived the critical period of ECM development without any evidence for neurological impairment (**Fig. 9A and B**). Anti-LFA-1 treatment had a significant protective effect on the integrity of the BBB (**Fig. 9C**). Formamide extraction of brains from Evans blue inoculated mice revealed that compared with the group of PbA-infected mice (N = 7; 6 ECM-positive and 1 ECM-negative), the group of anti-LFA-1 treated mice (N = 8; 1 ECM-positive and 7 ECM-negative) exhibited significantly less vascular leakage into the brain parenchyma. Separate analysis of the ECM-positive and ECM-negative mice within these two groups of mice revealed that preservation of BBB integrity prevented ECM development (**Fig. 9C**). This result again confirms that vascular leakage and neurological signs are directly correlated. Graphic depiction of the parasitemia-to-RMCBS ratio emphasizes the effect of LFA-1 treatment on ECM development (**Fig. 9D**). Application of two doses of anti-LFA-1 antibody on day 5 and 7 post infection extended the survival of 3/3 PbA-infected CBA mice to day 9; IVM of these mice provided no evidence for ECM (data not shown). Thus, blockage of LFA-1 mediated cellular interactions before manifestation of neurological signs prevents neuroimmunological BBB opening in a dose-dependent fashion.

### Platelet behavior and fibrin deposition

**Figure 10:**
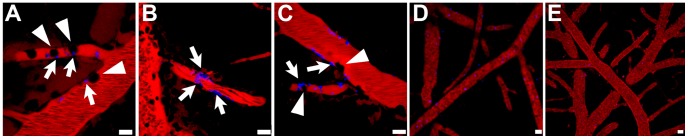
Platelet arrest correlates with ECM, not HP. CBA/CaJ mice were infected with PbA-GFP or PyXL-RFP iRBC and subjected to craniotomy at the onset of neurological signs or at >50% parasitemia, respectively. A–C) In PbA-infected mice, individual platelets or small aggregates (arrows) adhered to the endothelium of capillaries or PCV and remained stationary during the entire time of recording. Several blood cells (black, arrowheads in A) become arrested in a capillary suggesting that platelet deposits can act as foci for leukocyte arrest and temporary obstruction. D) In PyXL-infected mice, platelets (blue) circulate in large numbers at bloodstream velocity without adhering to the vascular endothelium. E) In uninfected control mice, platelets travel at bloodstream velocity and do not arrest. Scale bars = 10 µm. **Video S9, S10, S11, S12.**

Platelets are crucially involved in the early phase of ECM and selective platelet elimination was shown to prevent ECM [88]. In agreement with this notion, IVM revealed that individual and small clusters of platelets were marginalized in capillaries and PCV from mice with ECM (**Fig. 10A–C** **, Video S 9, 10, 11**). In striking contrast, platelets traveled at bloodstream velocity during at least one hour of intravital examination in PyXL-RFP-infected mice with HP (**Fig. 10D** **, Video S 12**) and in uninfected control mice (**Fig. 10E**) and failed to arrest. Antibody-mediated blockage of LFA-1 on day 5 after PbA infection strongly reduced platelet marginalization at the time when ECM would normally develop (day 6–8; data not shown). Thus, ECM, but not HP, correlates with focal platelet deposition in the cortical microvasculature, whose endothelial surface is normally non-thrombogenic.

Because severe *P. falciparum* malaria can be associated with focal disturbances of the coagulation system [89], [90] and coagulation factors have been observed to accumulate along the inner lining of venules, but not arterioles [91], we inoculated mice with fluorescent fibrinogen prior to IVM. Small precipitates of fibrin (or fibrinogen) were detected inside the PVS of morphologically intact PCV (**Fig. S7A**). As above, only a few platelets were associated with these fibrin-positive PCV. Occasional microvessels that appeared blocked or injured exhibited patches of perivascular fibrin and a small number of platelets (**Fig. S7B**). Overall, the amount of perivascular fibrin was modest and did not seem to extend beyond the PVS. In contrast, mechanical damage artificially inflicted during surgery caused widespread fibrin deposition in the PVS and infiltration of the brain parenchyma (**Fig. S7C**). No extravascular fibrin was found in uninfected control mice (**Fig. S7D**). Thus, consistent with the highly focal intravascular accumulation of platelets (**Fig. 10**) and the absence of major microvascular injury, ECM is associated with leakage of moderate amounts of fibrinogen across the PCV endothelium into the PVS.

### Role of LFA-1 in ECM-associated leukocyte recruitment

Because platelets are involved in endothelial activation and leukocyte recruitment [37], [85], [92], [93] and anti-LFA-1 treatment was shown to prevent ECM by selective abrogation of platelet sequestration [88], we examined the effect of antibody-mediated blockage of LFA-1 on leukocyte accumulation in the brain of groups of 6 PbA-infected CBA mice. Group 1 was imaged upon ECM development on day 6. Group 2 was treated with anti-LFA-1 on day 5 and group 3 was treated on day 5 and 7; both groups were subjected to IVM on day 7 after infection. Prior to IVM, all mice were inoculated with Evans blue, which allows detection of (negatively stained) leukocytes in the vascular lumen. Compared to the untreated control, the number of leukocytes decreased by 34% (Student's t-test: *P*<0.01) and 80% (*P*<0.001) in response to one and two anti-LFA-1 treatments, respectively (**Fig. S8**). Thus, in agreement with the inhibitory effect of anti-LFA-1 on platelet adhesion and manifestation of ECM, blockage of LFA-1 mediated cellular interactions abrogated leukocyte sequestration in and vascular leakage from cortical PCV (**Fig. 9**).

**Figure 11:**
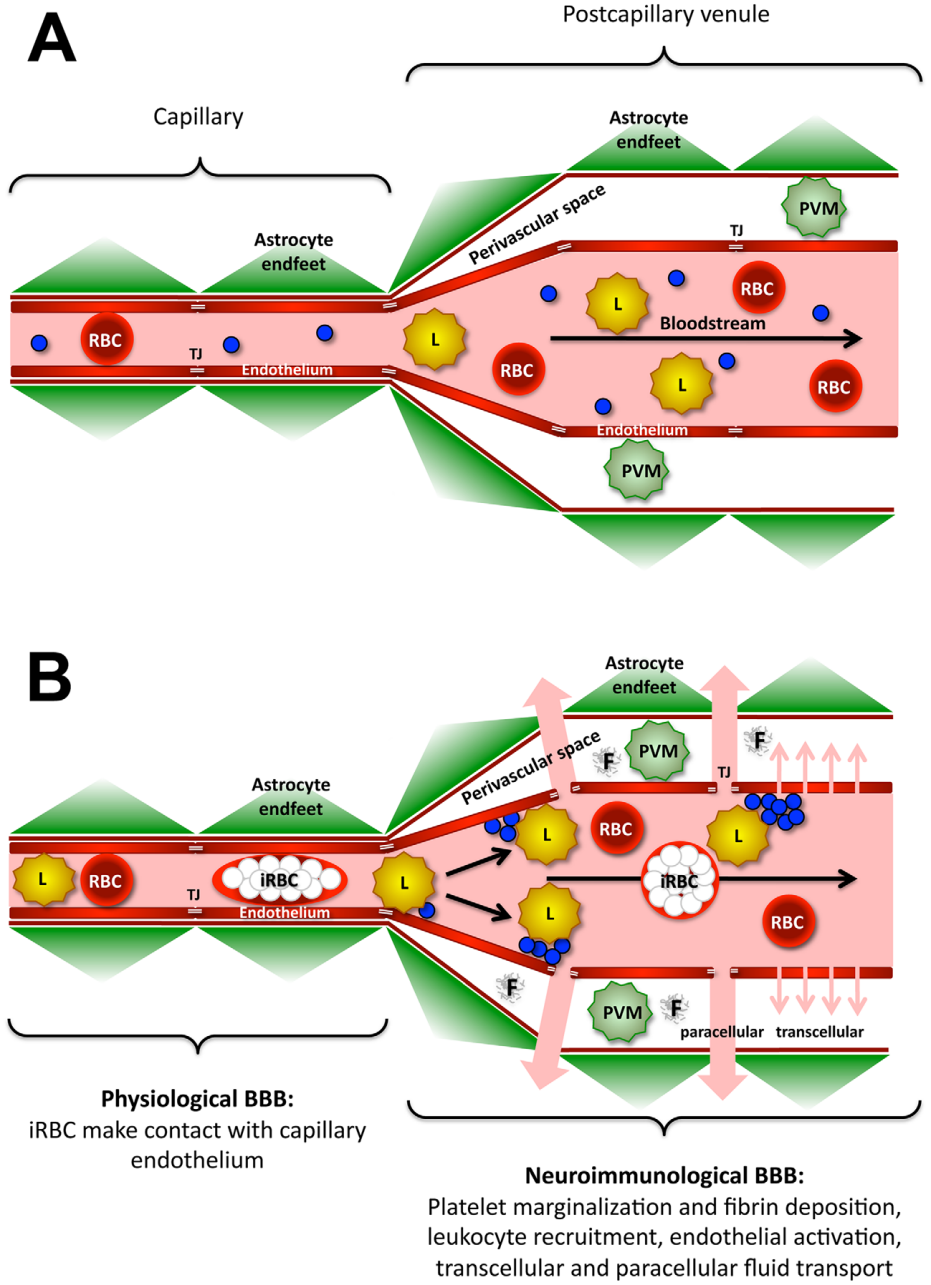
Model for the response of the two BBB to ECM. A) The cerebral microvascular tree contains two functionally distinct BBB. The physiological BBB is formed by capillaries (diameter 4–8 µm) and consists of a single layer composed of endothelia, gliovascular membrane, and astrocyte endfeet. The neuroimmunological BBB is formed by postcapillary venules (diameter 10–60 µm) and encompasses two layers that are separated by the perivascular space: 1) the endothelium with its basement membrane and 2) the glia limitans with associated astrocyte endfeet. While the physiological BBB serves as a diffusion barrier for solutes, the neuroimmunological BBB is characterized by higher permeability, which allows passage of macromolecules and diapedesis of immune cells into the perivascular space [69]. B) Although iRBC come into close contact with capillary endothelia, the physiological BBB remains intact during ECM. iRBC do not bind to the wall of PCV, but platelet marginalization and leukocyte recruitment indicate that PCV endothelia become activated in response to the infection. Leukocyte adhesion to and crawling along the endothelium involve LFA-1/ICAM-1 mediated cellular interactions. Eventually, the neuroimmunological BBB opens, initially in a reversible fashion, thus allowing the leakage of plasma into the PVS. The fluid transport is regulated and occurs via paracellular-junctional (large arrows) and/or transendothelial-vesicular (small arrows) pathways in the absence of cell death. PVM = perivascular macrophage, L = leukocyte, blue circles = platelets.

Taken together, our observations support the notion that ECM, but not HP, in PbA-infected young CBA/CaJ mice correlates with vascular leakage, platelet marginalization, deposition of fibrin in the PVS, and selective impairment of the neuroimmunological, but not the physiological BBB, throughout the cerebral and cerebellar parenchyma (**Fig. 11**). Death from ECM is preventable by blockage of LFA-1 mediated cellular interactions and by modulation of transcellular and paracellular pathways of transendothelial fluid transport.

## Discussion

A major finding of this study is that PbA and PyXL iRBC do not cytoadhere to the cerebral endothelium, but do occasionally become trapped in occluded microvessels thus explaining the moderate increase in parasite biomass in the brain [54], [94]. More importantly, neurological signs and death from ECM correlated with vascular leakage from PCV, not arterioles or capillaries, suggesting that ECM is associated with dysfunction of the neuroimmunological, but not the physiological BBB. Oral treatment of mice with imatinib and FTY720, drugs that were implicated in the modulation of transcellular-vesicular and paracellular-junctional fluid transport pathways, respectively [83], [84], prevented vascular leakage indicating that BBB opening is regulated. Cerebral microhemorrhages were also seen in ECM, but the relatively rare occurrence of these focal injuries argues against a major contribution to the observed widespread and rather uniform vascular leakage (**Figs. 4** **–** **7** **and S2, S4, S5, S6, S7, S8, S10**). PbA infection caused the most significant damage to the BBB of CBA/CaJ mice and also, albeit to a lesser degree, of SW mice. Because PbNK generally fails to induce neurological signs in mice, it has been used as a negative control for PbA induced ECM [31], [52], [80]–[82]. However, this parasite did cause BBB breakdown in the 3 week-old CBA/CaJ mice used here. The PbNK-induced ECM susceptibility may be age-related, since young, but not older, rats were also reported to develop a neurological syndrome [47]. None of the PyXL- or PyYM-infected mice examined here exhibited any evidence for neurological impairment or vascular leakage despite HP and consequently exposure of the cerebral microvasculature to significantly larger numbers of iRBC compared to PbA- or PbNK-infected mice that did develop ECM.

The exact mechanism of disruption of the human BBB and the pathological consequences including cerebral edema in HCM has long been unclear [95]–[97]. The correlation between the number of hemorrhages and poor clinical outcome, the highly focal nature of immune cell and platelet accumulation in the brain, the relatively rare occurrence of perivascular inflammation and hemorrhages, and the apparent absence of gross leakage of plasma proteins led to the proposal that BBB disruption is due to endothelial apoptosis caused by CD8+ CTL attack and granzyme B and perforin-mediated cytotoxicity [39], [53], [55], [98]. Endothelial apoptosis is also induced by *P. falciparum* iRBC, cytokines such as TGF-β1, platelets, and soluble parasite factors *in vitro* [99], [100]. According to this scenario, BBB disruption would be due to focal denudation of the vascular wall following endothelial cell death. However, our IVM studies of ECM provide no evidence for endothelial death or significant microvascular injury other than that associated with microhemorrhages. Neither the apoptosis marker YOPRO-1 nor the dead cell marker propidium iodide, which we used in previous IVM work to identify necrotic cells [57], indicated no general loss of cell viability or discontinuities in the vascular lining. These findings suggested that two different mechanisms of vasculopathy operate in parallel, albeit to different degrees, in the pathogenesis of ECM and HCM: neuroimmunological BBB opening via transendothelial fluid transport in the absence of injury and hemorrhaging due to endothelial death.

Modulation of the S1P pathway with FTY720 and inhibition of vesicular transport with imatinib prevented vascular leakage and ECM suggesting that the ECM-associated opening of the neuroimmunological BBB involves a combination of paracellular-junctional and transcellular-vesicular pathways [101]. This notion is in agreement with earlier work showing that endothelial barrier stabilizing mediators such as sphingosine-1-phosphate (S1P) and angiopoietin-1 (Ang-1) regulate reversible paracellular and/or transcellular fluid transport mechanisms by opposing the action of permeability-increasing mediators such as platelet-activating factor (PAF), bradykinin, thrombin, histamine, and VEGF [101], [102]. However, FTY720 also inhibits T cell egress from lymph nodes, changing the dynamics of leukocyte recruitment to the brain, and imatinib is also known to directly affect T cell function [103], [104]. Studies are underway to determine if FTY720 and imatinib prevent ECM by modifying the immune response within the brain, rather than by directly effecting endothelial cell function, whether junctional integrity or vesicular transport. Whatever the exact mechanistic basis of the ECM-associated vascular leakage, the S1P pathway was shown to be associated with protection against ECM, and Ugandan children with HCM exhibited decreased S1P plasma levels compared to those with uncomplicated malaria [84]. Further, low Ang-1 and high Ang-2 plasma levels are associated with retinopathy, discriminate HCM and severe non-cerebral from uncomplicated malaria, and predict mortality from HCM in Malawi and Central India [105]–[108]. Together, these findings indicate that the CM-associated BBB opening is preventable and suggest the possibility that BBB opening in humans and mice is regulated by similar mechanisms. Thus, endothelial barrier-stabilizing mediators such as FTY720, an FDA-approved drug for oral treatment of multiple sclerosis [109], may represent a novel class of adjunctive therapeutics for patients with HCM [84].

Vascular leakage rapidly intensified towards the final stages of neurological impairment (RMCBS = 3–7) and brains from mice with symptomatic ECM were clearly swollen. Evans blue leakage was observed to occur from PCV, i.e. a known site of immune cell sequestration and extravasation [69], but not from capillaries. The recent report that the physiological BBB serves as a tight diffusion barrier for small solutes, while the neuroimmunological BBB permits transport of macromolecules and diapedesis of immune cells [69], indicates that endothelia from capillaries and PCV contribute differently to the maintenance of BBB integrity and highlights the functional complexity of the cerebral microvasculature. Interestingly, PbA infection is associated with constriction of the cortical microvasculature [68], [110] and vasoconstriction can lead to transient opening of endothelial tight junctions [111], [112]. Therefore, it seems likely that BBB dysfunction in ECM occurs in the absence of endothelial cell death. ECM coincided with deposition of small amounts of fibrin (or fibrinogen) into the PVS of morphologically intact PCV with normal blood flow, further supporting our hypothesis that dysfunction of the neuroimmunological BBB during ECM is independent of major microvascular injury. This notion is strengthened by *in vitro* studies demonstrating that *P. falciparum* iRBC cause tight junction opening via trypsin-resistant molecules expressed on the iRBC membrane in combination with a soluble factor that is released at the mature trophozoite to schizont stages, leading to BBB opening before iRBC rupture with release of merozoites, hemozoin and other potentially inflammatory components [113], [114]. The PCV-associated vascular leakage seen in ECM is in accordance with the observation that in HCM, *P. falciparum* iRBC sequestration occurs predominantly within PCV [115], [116]. Further, a regulated mechanism of vascular leakage, i.e. BBB impairment in the absence of endothelial cell death, may explain why treatment with anti-malarial drugs at the onset of HCM rapidly reverses neurological signs and allows swift recuperation. While earlier studies reported that 88% of the pediatric and 97–99% of the adult patients surviving HCM have no residual neurological sequelae, suggesting that recovery from coma after treatment is complete in most cases [117], [118], and estimated that 80% of children recover from the cytokine storm caused by cerebral iRBC sequestration and formation of ring hemorrhages [12], recent work using more sensitive tests indicates higher post-HCM deficits [4]. Nevertheless, clinical symptoms after anti-malarial treatment are often completely and rapidly reversible suggesting that HCM may involve widespread, regulated and initially reversible, opening of the neuroimmunological BBB via transcellular-vesicular and paracellular-junctional pathways. Tight junction opening may conceivably expose matrix components such as collagen and von Willebrand factor that might trigger the platelet accumulation and coagulation observed in ECM. Interestingly, IVM revealed that CD14 was expressed on the luminal surface of endothelia from PCV, but not arterioles or capillaries, from PbA-infected mice with ECM, but not PyXL-infected mice with HP (**Fig. S5**). In accordance with the involvement of CD14 in endothelial activation, neuroinflammation, and leukocyte recruitment to cerebral PCV [66], [67], [119], [120] and the dominant role of this pattern recognition receptor in the induction of ECM [40], we found that the neuroimmunological BBB is also the site of arrest of CD8+ T cells, neutrophils, and monocytes during ECM (Movila, Nacer, and Frevert, manuscript in preparation). Vascular leakage occurred in all major organs of ECM mice including lungs, heart, kidneys, intestines, and skin (Nacer and Frevert, unpublished data) suggesting that loosening of tight junctions could account for the multiple organ failure occasionally observed in HCM. Studies are underway to investigate the contribution of CD14 to the observed opening of the neuroimmunological BBB.

Blockage of LFA-1 (CD11a) with a single dose of antibody on day 5, the day prior to the onset of ECM, prevented neurological signs and plasma leakage into the brain parenchyma in 6/10 mice (Kaplan-Meier log rank analysis: *P*<0.001) (**Fig. 9**). Further, a single dose of anti-LFA-1 antibody on day 6, but before the onset of ECM development (RMCBS = 13–14), also prolonged survival from day 6 to day 9 (data not shown). In agreement with the notion that platelets, which express LFA-1 on their surface, are crucially involved in the early stages of the pathogenesis of both ECM and HCM [37], [121], [122], Grau and colleagues reported that a single dose anti-LFA-1 antibody, when inoculated at the time of infection, was sufficient to protect mice from ECM, whereas multiple inoculations (on day 6, 8, and 10) were required for complete protection when treatment was started one day prior to ECM development [86]. Anti-LFA-1 treatment after manifestation of clear neurological signs did not alter the course of disease development in our hands. Thus, blockage of LFA-1 prevents ECM development in a time and dose-dependent manner. Insight into the underlying mechanism comes from the observation that prevention of ECM via LFA-1 blockage correlated with the selective abrogation of platelet sequestration in the brain [88] and that suppression of the PbA-induced LFA-1 expression in protein kinase C theta-deficient mice diminished the ability of CD8+ T cells to sequester in the brain resulting in a decrease in neurological signs [123]. Together with our finding that ECM correlates with focal platelet deposition along the microvascular endothelium and that blockage of LFA-1 inhibits leukocyte arrest in PCV from PbA-infected mice, these data confirm the crucial role of platelets in leukocyte recruitment to sites of infection [85], [92], [93].

ICAM-1 (CD54), an endothelial ligand of LFA-1, is constitutively expressed in low concentrations in the microvasculature, but greatly upregulated upon stimulation by pro-inflammatory cytokines such as TNF-α [85], [92]. Further, infection of the endothelium with Semliki Forest virus caused infiltration of the CNS by LFA-1^+^/Mac-1^+^ CD8+ T cells followed by a local increase in ICAM-1 expression and BBB breakdown [124]. ICAM-1 is thought to play a critical role in the pathogenesis of both ECM and HCM [92], [125], [126]. In ECM, ICAM-1 expression on brain endothelial cells was reported to increase on day 6 post infection, coinciding with a significant increase in microvascular permeability [86], [127]. Accordingly, late anti-LFA-1 treatment prevented PbA mortality [86], [87] and ICAM-1 deficient mice were protected from ECM [127]. Conversely, antibody-mediated ICAM-1 ligation caused the rapid death of PbA-infected, but not uninfected mice [86]. In HCM, ICAM-1 is upregulated and involved in the PfEMP-1 mediated cytoadherence of *P. falciparum* iRBC [85], [92], [125], [126]. Interestingly, a polymorphism in the N-terminal domain of ICAM-1 that is present with high frequency in African populations is associated with susceptibility to HCM [128]. ICAM-1 signaling is involved in endothelial activation, rearrangement of the endothelial actin cytoskeleton, regulation of vascular permeability, and transmigration of T cells through endothelial tight junctions into the brain parenchyma under various other inflammatory conditions [85], [92], [93], [129]. Further, *P. falciparum* iRBC rolling on ICAM-1 increases subsequent cytoadherence to CD36 [130]. For these reasons, it seems plausible that ICAM-1 signaling contributes to the opening of the neuroimmunological BBB. Because *P. vivax* also appears to export proteins to the iRBC surface that are able to interact with endothelial ICAM-1 [131], this signaling mechanism may account for the pathology not only in *P. falciparum* HCM, but also in *P. vivax* HCM and PbA ECM, despite the fact that the two latter parasites do not display significant sequestration in the brain.

None of the parasites studied here accumulated to any significant degree in the cortical microvasculature of any of the mouse strains examined. Under dynamic conditions, PbA and PyXL iRBC failed to adhere to the cortical vascular endothelium of mice with symptomatic ECM and HP, respectively. iRBC that remained stationary during IVM appeared to be trapped in vessels blocked by arrested leukocytes. In agreement with various earlier reports [13], [19], [33], our tissue sections from exsanguinated ECM mice failed to demonstrate any significant level of iRBC accumulation in the brain. Instead, the concentration of intravascular iRBC reflected the parasitemia at the time of sacrifice. Nevertheless, our dynamic *in vivo* observations do show that PbA iRBC harboring late-stage parasites come into close contact with capillary endothelia. Because PbA infection is associated with vasoconstriction, vascular obstruction, and ischemia [110], [132], the transient intimate contact between PbA iRBC and capillary endothelia could trigger pathogenetic mechanisms equivalent to those elicited by truly sequestered *P. falciparum* iRBC in HCM [1], [16], [126], [133], [134]. Despite the lack of knobs on PbA iRBC, it is entirely possible that other parasite antigens or secreted factors mediate BBB opening. Alternatively, transient interactions may provide pathogenic signals during HCM development as well. In agreement with the notion that *P. falciparum* iRBC sequestration does not lead to microvascular occlusion [10], transient binding of *P. falciparum* iRBC may occur in capillaries and true cytoadhesion in PCV, i.e. in distinct compartments of the microvasculature. Although iRBC sequestration in the cerebral microvasculature is generally considered a central factor in HCM pathogenesis, other mechanisms must clearly exist, because parasite sequestration is also found in patients dying from other complications of *P. falciparum* malaria [135]. Considering that *P. falciparum* iRBC sequestration in the brain can occur without vascular alterations [5], the contribution of cytoadherent iRBC to HCM development remains to be elucidated.

*Plasmodium* infection introduces marked structural changes on the erythrocyte membrane, leading to cellular distortion and decreased deformability in mature iRBC [136]–[138]. Because infected cells do not become enlarged as a result of the presence of a parasite [139], we speculate that increased rigidity [140] is responsible for the reduced velocity of mature iRBC in cerebral capillaries. This observation is particularly relevant for *Plasmodium* species with propensity for reticulocytes because of the larger size (8.6 µm) of these erythroid precursor cells compared to mature erythrocytes (7.6 µm) [141]. The large size of the slow moving iRBC we observed in cortical capillaries of SW mice with ECM compared to non-infected RBC is in agreement with the 150-fold preference of PbA for reticulocytes over normocytes [142]–[149]. Similar to our *in vivo* observations, the speed of *P. vivax* infected reticulocyte passage through narrow ducts is also inversely correlated with parasite maturity [150]. However, because slow-moving PbA-, PbNK-, or PyXNL-infected reticulocytes were not detected in CBA/CaJ or Balb/c mice, we cannot exclude other causes for the reduction in iRBC velocity in SW mice such as transient vascular obstruction by crawling leukocytes. Thus, we are only beginning to understand why, for example, PbA-, but not PbNK-infected reticulocytes induce ECM in susceptible mice and why CBA/CaJ mice respond to PbA infection with ECM and DBA mice with acute lung injury [151].

Although some options have become available to study the blood flow alterations associated with HCM, for example orthogonal polarization spectral imaging of the rectal or sublingual mucosa [152], [153], *in vivo* microscopy of the human brain is not possible. However, careful interpretation of static microscopic data does provide some insight into dynamic events. For example, based on the observation that the different developmental blood stages of *P. falciparum* are focally clustered and segregated by age within individual cerebral microvessels, Silamut and colleagues proposed that adherent iRBC do not detach or re-enter the circulation before maturation is complete and that cytoadherent iRBC do not occlude microvessels or block the blood flow [10]. The correlation between retinal pathology and malarial encephalopathy allows monitoring of disease progression [154], [155], but this approach is hampered by a certain degree of misclassification [12] and lacks the resolution required to observe the dynamics of iRBC, leukocyte, and platelet sequestration in the microvasculature; the mechanism of capillary occlusion; and the sequence of these events. Thus, ophthalmological and postmortem studies are very valuable, but provide limited information regarding the spatio-temporal progress of parasitological and pathological processes in HCM patients. Small animal models also have limitations, because the host/parasite pairs are different, but they are invaluable for study of the dynamics of ECM pathogenesis [15], [27], [156], provided results are correlated with available knowledge of HCM.

In conclusion, we propose a model for ECM, in which iRBC do not cytoadhere to or directly injure the endothelium of the cerebral microvasculature. LFA-1/ICAM-1 mediated cellular interactions promote platelet marginalization and leukocyte sequestration in PCV downstream from the capillary bed (**Fig. 11**). While the physiological BBB remains intact, regulated fluid transport across the neuroimmunological BBB leads to brain swelling, intracranial hypertension, coma, and ultimately death due to alteration of respiratory centers in pons and medulla oblongata as a result of brain stem compression. HCM may conceivably occur in two steps as it involves considerably more micro-hemorrhaging compared to ECM: 1) induction of coma based on regulated, and therefore preventable and reversible, opening of the BBB and 2) hemorrhaging due to endothelial death, which is difficult to reverse by treatment and eventually fatal. The existence of a regulated mechanism of neuroimmunological BBB opening would explain why coma is so rapidly reversible with treatment and with very little demonstrable tissue necrosis despite the large number of parasites in the brain of most patients, why some children have a poor neurological outcome while others improve with hardly any deficits despite similar clinical presentation, and how a largely intravascular parasite can cause so much neuronal dysfunction in HCM [4].

## Materials and Methods

### Parasites

The *P. berghei* and *P. yoelii* parasites were maintained by passage through female *Anopheles stephensi* mosquitoes [157]. *P. berghei* strains were wt PbA (non-fluorescent), green fluorescent PbA (PbA-GFP), a kind gift from Dr. Andy Waters, University of Glasgow, UK [158], wt *P. berghei* NK65 (PbNK), and GFP-expressing *P. berghei* NK65 (PbNK-GFP). *P. yoelii* strains were PyXL, originally derived from the non-lethal 17× strain [72], and PyYM, which were kindly provided by Drs. James Burns (Drexel University College of Medicine) and David Narum (Malaria Vaccine Development Branch, NIAID), respectively [148], [159]. These wt parasites were transfected to express RedStar, an improved version of the red fluorescent protein drFP583/DsRed/RFP [160], under the control of the elongation factor 1α promoter using a novel replacement strategy [161], [162] (termed PyXL-RFP and PyYM-RFP, respectively). Briefly, the RFP expressing cassette was stably integrated in S1 (sporozoite expressed gene 1) locus (PY05712) of PyXL and PyYM via double crossover homologous recombination. The S1 locus is dispensable in *P. yoelii* genome and can be used for stable transgene expression throughout the parasite life cycle [163]. All green or red *P. yoelii* and *P. berghei* strains emit fluorescence throughout the entire life cycle.

### Ethics statement

This study was conducted in strict accordance with the recommendations in the Guide for the Care and Use of Laboratory Animals of the National Institutes of Health. The protocol was approved by the Institutional Animal Care and Use Committee, NYU School of Medicine (Protocol number 101201-01). All surgery was performed under ketamine/xylazine/acepromazine anesthesia, and all efforts were made to minimize suffering.

### Mouse strains

Mice were Balb/c (Taconic, Germantown, NY), Swiss Webster (SW; Charles River, Wilmington, MA, and Taconic Farms, Inc., Germantown, NY), CBA/CaJ (Jackson Laboratory, Bar Harbor, ME), and A/J (Jackson Laboratory). CX3CR1-EGFP mice (B6.129P-Cx3cr1tm1Litt/J) were a kind gift from Drs. Dan Littman and P'ng Loke, NYU School of Medicine. Animals were maintained and used in accordance with recommendations in the guide for the Care and Use of Laboratory Animals. At the time of the experiments, the mice were 3 weeks old (body weight of 12–15 g) unless stated otherwise. Young mice were infected by intraperitoneal injection of 1×10^6^ iRBC. In agreement with other reports showing that both dose and mode of inoculation influence onset and speed of manifestation of the disease [72], [164], [165], PbA infection in older mice was lethal 6–7 days after intravenous inoculation of 1–5×10^6^ iRBC and 7–9 days after intraperitoneal inoculation of 5×10^5^ iRBC. The parasitemia was monitored daily using Giemsa stained blood smears and mice were sacrificed upon development of ECM or HP.

### Assessment of neurological signs

Neurological signs were assessed using the Rapid Murine Coma and Behavioral Scale [70]. Mice were considered ECM positive when two or more parameters clearly indicated behavioral alteration including body position, spontaneous activity, startle response, tremor, gait, touch escape, and righting reflex [70], [166]. *P. yoelii* infected mice exhibited a hunched position, pale skin color, and an increased respiration rate when parasitemia levels reached levels around 80%, but failed to present typical neurological manifestations.

### Histopathology

Groups of 3 mice of the various strains were infected with the indicated parasites. Brains from infected and control mice were removed after exsanguination under anesthesia and fixed with 4% paraformaldehyde in PBS. Coronal slices were prepared at Bregma [76] using an acrylic brain matrix (Stoelting, Wood Dale, IL), embedded in paraffin, and processed for H&E staining at the Histopathology Core Facility of New York University School of Medicine. Photographs were taken with a Nikon DS-Fi1 camera and imported into Nikon NIS Elements F 3.0 software (Nikon Inc., Melville, NY).

### Blood brain barrier integrity

Because of its ability to bind to albumin, Evans blue was used as a marker for protein leakage to determine BBB opening [78], [79]. Mice were intravenously injected with 100 µl of a 1% solution in PBS of the plasma marker Evans blue at the indicated times after infection with PbA, PbNK, PyXL, or PyYM. Uninfected mice were used as controls. Three hours later, mice were exsanguinated under anesthesia. Brains were removed, split into the two hemispheres, and weighed. Formamide (500 µl per hemisphere) was added and the samples were incubated for 3 days at 4°C protected from light. After 3 days of formamide extraction, Evans blue absorption was measured at 620 nm (OD620) [167], [168] using a Multiskan Multisoft microplate reader (Labsystems, Helsinki, Finland).

### Anti-LFA-1 treatment

Vascular leakage and survival. Two groups of 10 three week-old female CBA-CaJ mice were inoculated with 200 µl PbA-infected blood of 1% parasitemia. On day 5 after infection, one group of mice received an intravenous injection of 200 µg rat anti-mouse LFA-1 (CD11a; clone M17/4, azide-free, BioLegend, San Diego, CA). Neurological signs were recorded daily and graded according to the RMCBS [70]. Giemsa-stained blood smears were examined daily to monitor the parasitemia. PbA-infected and PbA-infected/anti-LFA-1 treated mice were inoculated on day 6–8 and 9, respectively, with 100 µl of a 1% Evans blue solution and euthanized 3 h later for brain removal, formamide extraction, and OD620 measurement.

### Leukocyte sequestration

Groups of 6 CBA mice were infected with PbA and subjected to brain IVM 1) on day 6 upon ECM development, 2) on day 7 after a single anti-LFA-1 treatment on day 5, or on day 7 after two anti-LFA-1 treatments on day 5 and 7. Prior to imaging, mice were inoculated with Evans blue. Multiple time sequences were recorded for quantification of the number of arrested leukocytes in the PCV lumen. Depending on the experimental condition, 20–45 PCV were analyzed per mouse.

### Mechanism of endothelial fluid transport

Groups of 5 PbA-infected CBA/CaJ mice received 1) two daily oral doses of 150 mg/kg imatinib on days 5, 6, and 7 post infection [83], [169], 2) one daily oral dose of 0.3 mg/kg FTY720 starting one day before infection [84], or 3) no treatment. Imatinib and FTY720 were dissolved in water and diluted with PBS [170]. At the onset of neurological signs, mice were injected with Evans blue and examined by IVM. Other brains were removed for Evans blue extraction and OD620 measurement. Mice surviving the critical period of ECM development were analyzed on day 10.

### Organ photography

Whole brains from Evans blue injected mice were photographed with a Leica MZ16 FA stereomicroscope. Sagittal brain slices were prepared with acrylic matrices (Ted Pella, Redding, CA) and photographed accordingly. Data were imported into Adobe Photoshop Elements for further processing.

### Anesthesia and craniotomy

For IVM of iRBC dynamics and BBB opening, 6–8 week-old SW mice and 3 week-old CBA/CaJ mice, respectively, were infected by intraperitoneal inoculation of 0.5–10×10^6^ iRBC as indicated for the individual experiments. The parasitemia was monitored daily by Giemsa staining of blood smears. Upon appearance of neurological signs in PbA-infected mice or increase of the parasitemia to >50% in PyXL-infected mice (typically on day 5 or 6), the animals were anesthetized by intraperitoneal injection of a cocktail of 50 mg/kg ketamine (Ketaset, Fort Dodge Animal Health, Fort Dodge, IO), 10 mg/kg xylazine (Rompun, Bayer, Shawnee Mission, KS), and 1.7 mg/kg acepromazine (Boehringer Ingelheim Vetmedica, St. Joseph, MO) (KXA mix) as described [57], [58] and surgically prepared for intravital imaging of the brain [171]. After immobilization of the head in a stereotaxic apparatus (mouse and neonatal rat adaptor; Stoelting, Wood Dale, IL), the skull was exposed by midline incision of the skin and a cranial window 4–5 mm in diameter was generated using a high-speed microdrill (Fine Science Tools, Foster City, CA). After removal of the bone flap, the exposed Dura mater was kept moist with gelatin foam (Gelfoam, Pfizer Inc., New York) until the window was closed with a cover slip glued to the skull [172]. Respiration-induced movement of the brain was minimized by immobilization of the cover slip on a custom-made stainless steel adapter for the microscope stage.

### Intravital microscopy (IVM)

After measuring the thickness of the murine cerebral meninges in paraffin-embedded brain sections stained with H&E, Trichrome, and van Gieson (**Fig. S9 and Video S13**), we confirmed by confocal scanning that data acquisition was possible to a depth of 50 µm (**Fig. S10 and Video S14**). Thus, it is possible to penetrate the 10–15 µm thick meninges and to examine the underlying cortical microvasculature. Further, IVM of CX3CR1-EGFP mice with green fluorescent microglia confirmed that the microvessels analyzed in this study are located in microglia-rich, i.e. cortical, areas of the brain (**Video S8**).

Vascular leakage was visualized by intravenous injection of 20 µl of a 2 mM solution of bovine serum albumin (BSA) conjugated to FITC or Texas Red (Life Technologies, Grand Island, NY), 100 µl of a 1% Evans blue solution, or 50 µg Alexa Fluor 488 conjugated human fibrinogen (azide-free; Molecular Probes Inc., Eugene, OR). Vascular endothelia were labeled with intravenously injected Alexa 488 or eFluor 405-conjugated rat anti-mouse PECAM-1 (CD31; clone MEC13.3, BioLegend, San Diego, CA) and phycoerythrin (PE)- or Alexa 647-conjugated rat anti-mouse CD14 (clone Sa2–8, eBioscience, San Diego, CA). Vascular leakage from PCV was independent of fluorescent labeling thus ruling out the possibility that antibody binding to brain endothelia affected barrier function. Platelets were labeled by intravenous injection of 3–5 µg eFLUOR 405-conjugated rat anti-mouse CD41 (clone MWReg30, eBioscience). The cortical microvasculature was imaged with an inverted Leica TCS SP2 AOBS confocal system [57], [79], [173]. Microvessels at the center of the cranial window were chosen for imaging to avoid possible surgery-related artifacts along the perimeter of the craniotomy. The body temperature of the animals was stabilized with a temperature-controlled Ludin chamber attached to the microscope. Periodic subcutaneous reinjection of KXA mix allowed intravital microscopic examination of the animals for 1–2 hours. Appropriate laser lines were chosen and laser power was reduced to a minimum to avoid phototoxicity and bleaching. These optimized excitation conditions have allowed us to monitor live fluorescent parasites for a period of up to 6 h without any apparent effect on viability in previous studies [57], [58].

### Image processing

Confocal microscopy data sets were acquired with Leica Confocal Software. Image-Pro Plus (Media Cybernetics, Bethesda, MD), AutoDeBlur (Media Cybernetics, Bethesda, MD), and Imaris 7.2 (Bitplane, Saint Paul, MN) were used for further image analysis, deconvolution, and 3D reconstruction.

### Statistical analyses

Data were analyzed using Minitab version 15. Data sets were evaluated for normality and equal variances prior to testing with a one-way ANOVA or General Linear Model as appropriate, following transformation where data were not normally distributed [174]. Post-hoc analyses were performed using Dunnett's Test for comparisons with a control. Survival curves were generated with SigmaPlot 12 (Systat Software, Inc., San Jose, CA) and subjected to Kaplan-Meier log-rank analysis.

## Supporting Information

Figure S1 **Visualization of the blood velocity.** The fluorescent plasma markers BSA-TX (red in A) and BSA-FITC (green in B) were used to visualize the vascular lumen of SW mice infected with PbA-GFP (green in A) and PyXL-RFP (red in B), respectively. A) Note the high blood velocity in the lower and right branch of the vessel (arrows) compared to the slower flow in the upper branch (arrowheads). The relatively slow scan speed of the laser beam causes blood cells to appear distorted. With increasing blood velocity, iRBC (green) and RBC (dark) appear as ovals (arrowheads) to narrow streaks (arrows). Nuclei were stained with Hoechst (blue). B) Several slow-moving cells (arrows) flow from the right branch down into the larger vessel at low velocity. Shortly before the end of the time sequence, the velocity increases abruptly as indicated by the long dark streaks entering the larger vessel. The blood in the other two branches moves at high velocity. Scale bars = 10 µm. **Video S1 and S2.**
(TIF)

Figure S2 **PyXL-iRBC velocity is reduced in capillaries.** Several PyXL-RFP iRBC (red, arrows) travel at a reduced speed through the lumen of a capillary of PyXL-infected SW mice in this two-minute IVM recording, decreasing the velocity of other blood cells (dark). The vascular lumen is labeled with BSA-FITC. Scale bar = 10 µm. **Video S6.**
(TIF)

Figure S3 **Microvascular occlusions in PyXL-infected mouse brains.** A, B) A large number of iRBC (red) is present in an occluded larger microvessel, while the blood flow in a neighboring capillary is preserved (arrow). Note that BSA-FITC (green) is excluded from the blocked microvessel (arrowheads) indicating that vascular occlusion had occurred prior to injection of the fluorescent marker. B) Hemozoin reflection is shown in blue. Note that not all iRBC (red) contain hemozoin. Further, hemozoin is not always correlated with parasite fluorescence suggesting phagocytic uptake. C) Two blocked capillaries containing PyXL iRBC (red; arrowheads) run parallel to a larger blood vessel with preserved high-velocity blood flow (arrow). Nuclei were visualized with Hoechst (blue). Note the absence of BSA-FITC in the capillaries. Scale bars = 10 µm. **Video S7.**
(TIF)

Figure S4 **Time course of BBB opening during ECM development.** Groups of 3 CBA/CaJ mice were infected with PbA and injected with Evans blue 3 h prior to exsanguination and brain removal. A) Uninfected control, no leakage. B) Day 4 after infection: the unusually pale, whitish color of the brain suggests ischemia due to microvascular constriction. C) Day 5: the brain appears whitish with a blue tint. D) Day 6: massive Evans blue leakage indicates ECM with fully developed neurological syndrome.
(TIF)

Figure S5 **PCV and arterioles differ in CD14 and CD31 expression.** A) *In vivo* immunolabeling of a PbA-infected CBA/CaJ mouse with ECM reveals that CD31 (PECAM-1) is predominantly present on the luminal surface of arteriolar and capillary endothelia (green), while CD14 is expressed on venous endothelia (red). The projection of a 3D stack also shows that the level of CD14 expression is somewhat patchy within a given PCV. B) Brain from a PbA-infected CBA/CaJ mouse that had been inoculated with Evans blue (red) and PE-conjugated anti-mouse CD14 (green). In contrast to arterioles (white star), PCV are lined with CD14-labeled endothelia. C) Brain from a PyXL-infected mouse with HP that had been inoculated with Evans blue and PE-conjugated anti-mouse CD14 (green). While monocytes exhibit a CD14-positive surface label (arrow), PCV endothelia are CD14-negative. Note that the vascular marker has leaked into the parenchyma of the mouse brain infected with PbA (A), but not with PyXL (B). A) Maximum projection of a Z-stack, B and C) snapshots from time series. Scale bars = 20 µm.
(TIF)

Figure S6 **ECM is associated with vascular leakage throughout the brain.** A and B) PbA-infected CBA/CaJ mice with neurological signs or uninfected mice were injected with Evans blue. Three hours later, the brains were removed and sectioned coronally at Bregma. A) The blue shade of the sectioned brain surface indicates that Evans blue has widely infiltrated the cerebral parenchyma. B) In contrast, brains from uninfected control mice appear pink and due to a lack of Evans blue leakage into the tissue. C and D) Upon ECM development, a PbA-infected CBA/CaJ mouse was injected with Evans blue and PE-conjugated anti-CD14. Three hours later, the brain was removed for preparation of coronal vibratome sections. C) The maximum projection of a confocal Z-stack, which was taken from the center of the gray matter, reveals Evans blue (red) has leaked from a PCV lined with CD14-positive endothelia (green) into the gray matter as evidenced by red-stained neurons. D) In contrast, neither Evans blue leakage nor CD14 labeling is detectable in a brain vibratome section from an uninfected control mouse. Scale bars = 20 µm.
(TIF)

Figure S7 **ECM is associated with perivascular fibrin deposition.** A) PCV exhibit focal deposits of fibrin (or fibrinogen, green) within the PVS and occasional platelets (blue) in the absence of visible vascular injury. B) Rare sites of vascular injury are associated with aggregates of platelets (blue, arrow) and fibrin (green) deposits along the vascular wall, in the PVS, and in the parenchyma (arrowhead). The absence of luminal Evans blue (white star) indicates microvascular occlusion. C) Mechanical damage to the cortical microvasculature results in extensive fibrin leakage into the PVS and parenchyma (arrowheads). D) In uninfected control mice, fibrinogen remains uniformly distributed in the bloodstream and extravascular fibrin deposits were not detected. PbA-infected CBA/CaJ mice with ECM or uninfected control mice were inoculated with Alexa Fluor 488-conjugated human fibrinogen, platelets were detected with eFluor 401-conjugated anti-CD41. Bars = 50 µm.
(TIF)

Figure S8 **Anti-LFA-1 treatment reduces leukocyte recruitment.** A) At the time of ECM (day 6), a PCV from a PbA-infected CBA mouse contains a large number of arrested leukocytes (arrowheads). Leukocytes exclude Evans blue and appear dark. Note the extensive leakage of the vascular marker into the PVS and surrounding tissue. B) Considerably fewer leukocytes are present in a PCV from a mouse that was imaged 2 days after a single anti-LFA-1 treatment on day 5. Note the absence of vascular leakage. C) Arrested leukocytes are rare and vascular leakage is absent after two anti-LFA-1 treatments on day 5 and 7. IVM was done 4 h after the second treatment. D) Cortical PCV from uninfected mice do not contain arrested leukocytes. Scale bar = 20 µm. E) Compared to untreated PbA-infected CBA/CaJ mice with ECM (0), one (1) and two (2) anti-LFA-1 treatments inhibit leukocyte arrest significantly in a dose-dependent manner. The data reflect the mean number of leukocytes/mm^2^ ± STD counted in 20–45 PCV depending on the experimental condition. Significance was determined with Student's t-test.
(TIF)

Figure S9 **Thickness of murine meninges.** A) H&E-stained sections of paraffin-embedded brain tissue show that the murine cerebral meninges measure 10–15 µm in diameter (double arrow). Note that compared to the underlying cortex, the meninges are highly vascularized and rich in nuclei. B) Coronal vibratome section of live brain tissue from a CBA/CaJ mouse that had been injected with the vascular marker Evans blue (red) and the nuclear stain Hoechst (blue). Note that compared to the cortical microvasculature, the meningeal blood vessels contain considerably larger amounts of Evans blue; confocal microscopy. **Video S13.** Scale bars = 20 µm.
(TIF)

Figure S10 **Confocal microscopy allows IVM of the cortical microvasculature.** Individual frames from a confocal Z-stack, reaching from the Dura mater 50 µm into the cerebral cortex. Pial microvessels (white stars) are visible at a depth of 3.6 µm and 7.1 µm. Note the presence of meningeal collagen fibers (red autofluorescence, arrowheads) at a depth of 7.1 µm and 11.9 µm. Cortical arterioles (*in vivo* labeled with eFluor 405-conjugated anti-CD31, blue) and PCV (*in vivo* labeled with PE-conjugated anti-CD14, green) become visible at 13.4 µm and are fully in focus at a depth beyond 19.6 µm. Scale bar = 20 µm. **Video S14.**
(TIF)

Video S1 **Visualization of the blood velocity in PbA-GFP-infected mice.** The red fluorescent plasma marker BSA-TX was used to visualize the vascular lumen of mice infected with PbA-GFP (green). The relatively slow scan speed of the laser beam causes blood cells to appear distorted. With increasing blood velocity, iRBC (green) and RBC (dark) appear as ovals to narrow streaks. Note the high blood velocity in the lower and right branch of the vessel compared to the slower flow in the upper branch.
(AVI)

Video S2 **Visualization of the blood velocity in PyXL-RFP-infected mice.** The green fluorescent plasma marker BSA-FITC was used to visualize the vascular lumen of mice infected with PyXL-RFP (red). Several slow-moving cells (dark ovals) slowly enter the larger vessel from the branch on the right. Shortly before the end of the Video, the velocity increases abruptly as indicated by the long dark streaks entering the larger vessel. In the other branches of the vessel, the blood moves at high velocity throughout the entire recording.
(AVI)

Video S3 **iRBC make intimate contact with capillary endothelia.** Several early- and late-stage PbA-GFP iRBC travel through a capillary in the course of this **Video**. Note the elongated shape of the two large iRBC, which most likely contain trophozoites or schizonts. While iRBC containing large late-stage parasites squeeze only slowly through the narrow capillary lumen, presumably due to decreased flexibility, iRBC harboring small early-stage parasites travel at blood velocity. The vascular lumen is labeled with BSA-TX (red); nuclei are stained with Hoechst (blue).
(AVI)

Video S4 **PbA-iRBC are trapped in occluded microvessels.** A larger blocked vessel (top) contains PbA-GFP iRBC (green), while a neighboring capillary (bottom) displays normal blood flow. The vascular lumen is labeled with BSA-TX (red).
(AVI)

Video S5 **PbA-iRBC are trapped in occluded microvessels.** A large number of iRBC (green) are visible in a large branched vessel that is completely blocked (center), while the blood flow is preserved in a capillary (bottom middle) and a large vessel (upper right). The vascular lumen is labeled with BSA-TX.
(AVI)

Video S6 **PyXL-iRBC reduce the blood velocity in capillaries.** Several PyXL-RFP iRBC (red) travel through the lumen of a capillary. Note that the velocity of other blood cells (dark) is decreased while an iRBC containing a mature parasite and several multiply infected iRBC pass through the capillary (frames 0.00–1.63 s and 22.82–45.64 s, respectively). The vascular lumen is labeled with BSA-FITC (green).
(AVI)

Video S7 **Microvascular occlusion in PyXL-infected mouse brains.** Two blocked capillaries containing PyXL iRBC (red) run parallel to a larger blood vessel with preserved high-velocity blood flow. Note the absence of BSA-FITC (green) in the capillaries.
(AVI)

Video S8 **ECM is associated with vascular leakage throughout the brain parenchyma.** Upon ECM development, a PbA-infected CX3CR1-EGFP mouse was injected with Evans blue. Three hours later, the brain was removed for preparation of live coronal vibratome sections. The confocal Z-stack, which was taken from the center of the cerebral cortex, reveals widespread leakage of Evans blue (red) into the gray matter, which contains numerous green fluorescent microglia. Scale bar = 100 µm.
(AVI)

Video S9 **ECM correlates with platelet marginalization.** This time series shows a cluster of aggregated platelets (blue) that adhere to the wall of a PCV and remain stationary during the time of intravital recording. Note that the blood flow is preserved. The vascular lumen was labeled with Evans blue (red). Representative CBA/CaJ mouse that was infected with PbA-GFP iRBC and subjected to craniotomy at the onset of clear neurological signs (RMCBS = 3–7).
(AVI)

Video S10 **Platelet marginalization promotes leukocyte arrest.** This time series shows several leukocytes (black) that have become arrested in close association with marginalized platelets. The vascular lumen was labeled with Evans blue (red). Representative CBA/CaJ mouse that was infected with PbA-GFP iRBC and subjected to craniotomy at the onset of clear neurological signs (RMCBS = 3–7).
(AVI)

Video S11 **Platelet marginalization promotes leukocyte arrest.** This Z-stack shows marginalized platelets in close association with arrested leukocytes (black). The vascular lumen was labeled with Evans blue (red). Representative CBA/CaJ mouse that was infected with PbA-GFP iRBC and subjected to craniotomy at the onset of clear neurological signs (RMCBS = 3–7).
(AVI)

Video S12 **Platelets travel at bloodstream velocity in mice with HP.** CBA/CaJ mice were infected with PyXL-RFP iRBC and subjected to craniotomy at >50% parasitemia. Platelets (blue) circulate in large numbers at blood velocity without adhering to the vascular endothelium. Note the absence of vascular leakage and arrested leukocytes. The vascular lumen is visualized with Evans blue (red).
(AVI)

Video S13 **Meningeal and cortical microvasculature.** Confocal Z-stack through a vibratome section of live brain tissue from an uninfected CBA/CaJ mouse that had been injected with Evans blue (red) and the nuclear stain Hoechst (blue). Note that the meninges cover the cortex as a highly vascularized thin layer (top right). Microvessels of various sizes are visible throughout the gray matter. The coronal section was taken at Bregma. Scale bar = 100 µm.
(AVI)

Video S14 **IVM of the meningeal and cortical microvasculature.** The confocal Z-stack extends from the Dura mater 50 µm into the cerebral cortex. Pial microvessels are visible at a depth of 3–12 µm, while cortical arterioles and PCV are in focus at a depth of 15 µm and beyond. Scale bar = 20 µm.
(AVI)
