# Supplementary figures and images for: Neuroimmunological Blood Brain Barrier Opening in Experimental Cerebral Malaria
Source: PLoS Pathog. 2012 Oct 25;8(10):e1002982. doi: 10.1371/journal.ppat.1002982 (PMC3486917; doi:10.1371/journal.ppat.1002982)

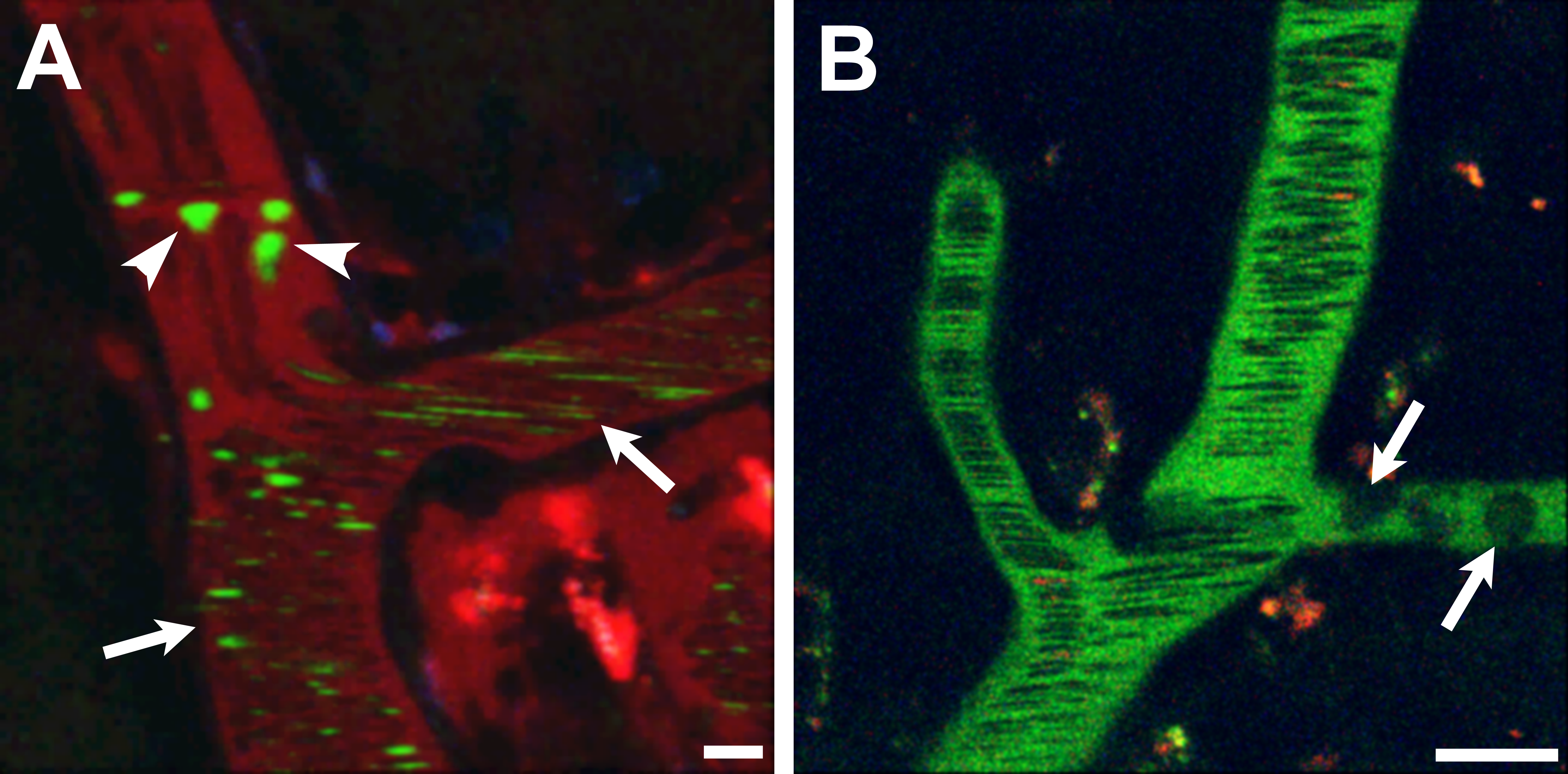

Supplement: Figure S1 — Visualization of the blood velocity. The fluorescent plasma markers BSA-TX (red in A) and BSA-FITC (green in B) were used to visualize the vascular lumen of SW mice infected with PbA-GFP (green in A) and PyXL-RFP (red in B), respectively. A) Note the high blood velocity in the lower and right branch of the vessel (arrows) compared to the slower flow in the upper branch (arrowheads). The relatively slow scan speed of the laser beam causes blood cells to appear distorted. With increasing blood velocity, iRBC (green) and RBC (dark) appear as ovals (arrowheads) to narrow streaks (arrows). Nuclei were stained with Hoechst (blue). B) Several slow-moving cells (arrows) flow from the right branch down into the larger vessel at low velocity. Shortly before the end of the time sequence, the velocity increases abruptly as indicated by the long dark streaks entering the larger vessel. The blood in the other two branches moves at high velocity. Scale bars = 10 µm. Video S1 and S2. (TIF) [file ppat.1002982.s001.tif]

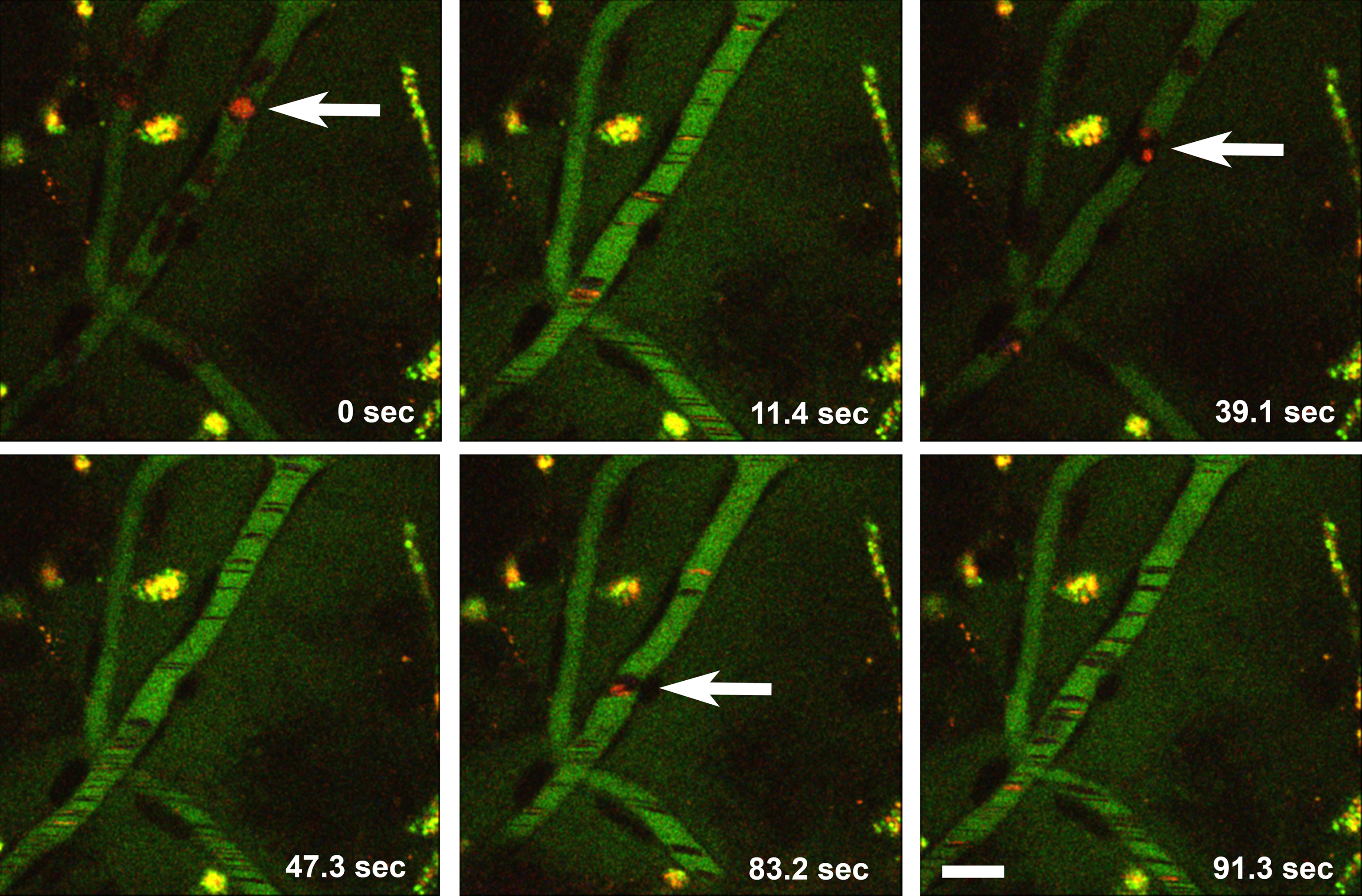

Supplement: Figure S2 — PyXL-iRBC velocity is reduced in capillaries. Several PyXL-RFP iRBC (red, arrows) travel at a reduced speed through the lumen of a capillary of PyXL-infected SW mice in this two-minute IVM recording, decreasing the velocity of other blood cells (dark). The vascular lumen is labeled with BSA-FITC. Scale bar = 10 µm. Video S6. (TIF) [file ppat.1002982.s002.tif]

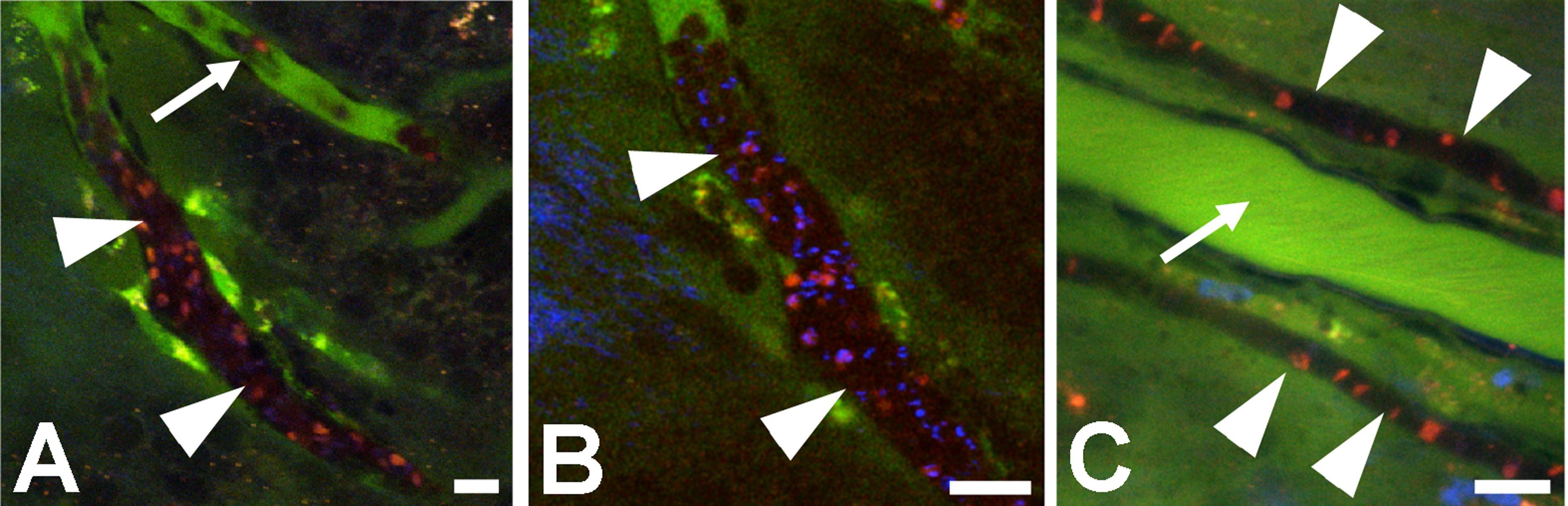

Supplement: Figure S3 — Microvascular occlusions in PyXL-infected mouse brains. A, B) A large number of iRBC (red) is present in an occluded larger microvessel, while the blood flow in a neighboring capillary is preserved (arrow). Note that BSA-FITC (green) is excluded from the blocked microvessel (arrowheads) indicating that vascular occlusion had occurred prior to injection of the fluorescent marker. B) Hemozoin reflection is shown in blue. Note that not all iRBC (red) contain hemozoin. Further, hemozoin is not always correlated with parasite fluorescence suggesting phagocytic uptake. C) Two blocked capillaries containing PyXL iRBC (red; arrowheads) run parallel to a larger blood vessel with preserved high-velocity blood flow (arrow). Nuclei were visualized with Hoechst (blue). Note the absence of BSA-FITC in the capillaries. Scale bars = 10 µm. Video S7. (TIF) [file ppat.1002982.s003.tif]

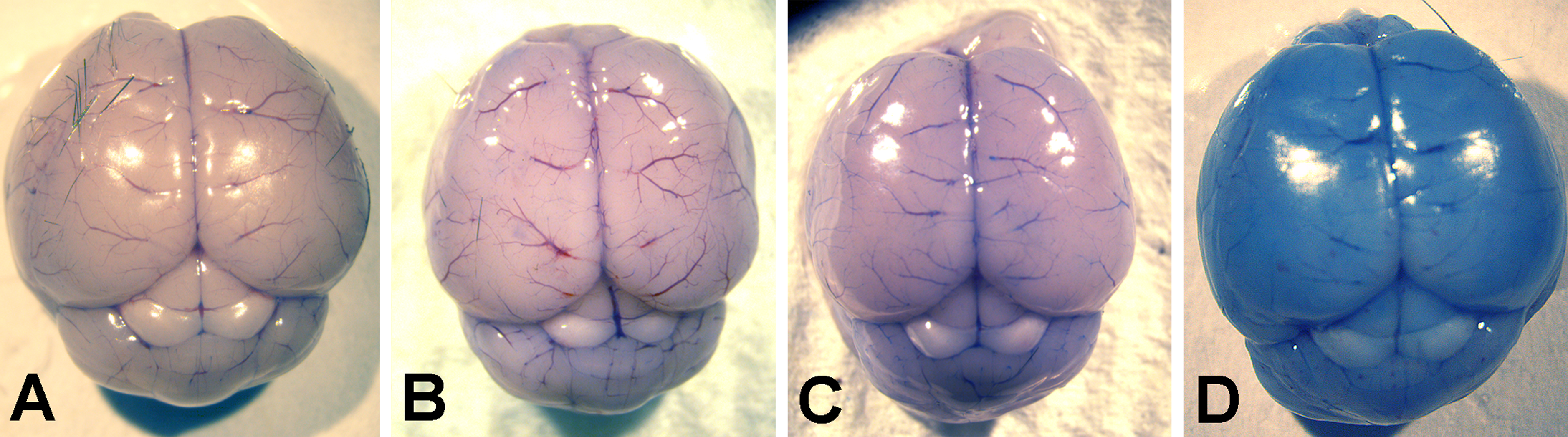

Supplement: Figure S4 — Time course of BBB opening during ECM development. Groups of 3 CBA/CaJ mice were infected with PbA and injected with Evans blue 3 h prior to exsanguination and brain removal. A) Uninfected control, no leakage. B) Day 4 after infection: the unusually pale, whitish color of the brain suggests ischemia due to microvascular constriction. C) Day 5: the brain appears whitish with a blue tint. D) Day 6: massive Evans blue leakage indicates ECM with fully developed neurological syndrome. (TIF) [file ppat.1002982.s004.tif]

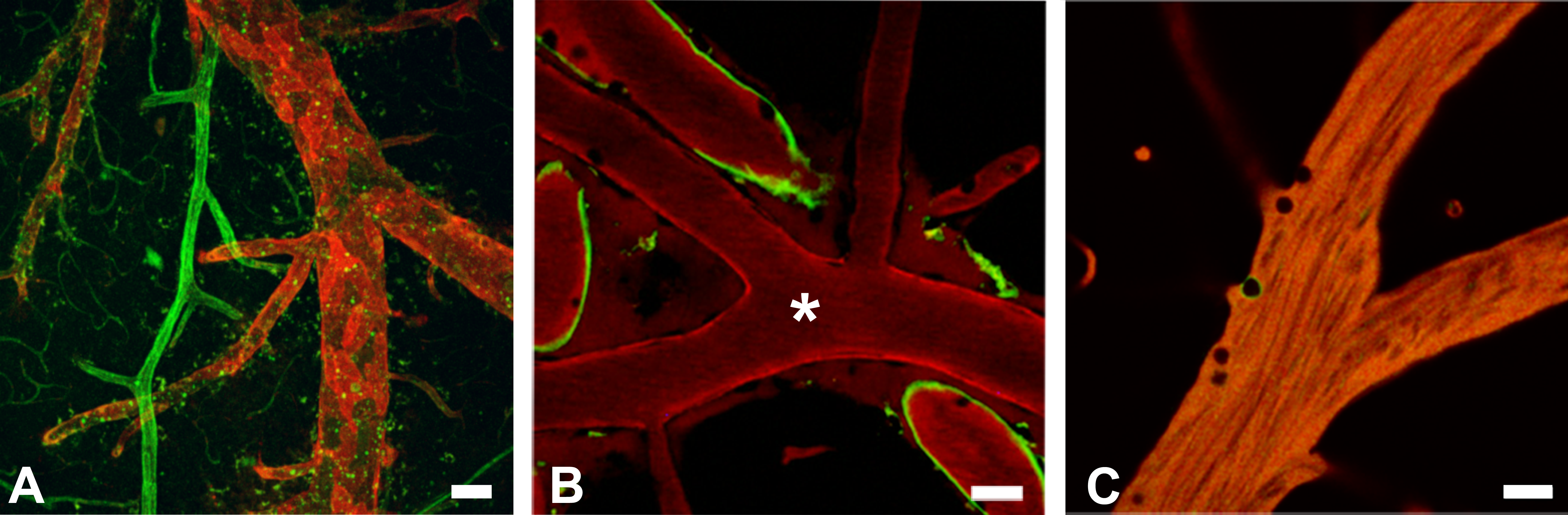

Supplement: Figure S5 — PCV and arterioles differ in CD14 and CD31 expression. A) In vivo immunolabeling of a PbA-infected CBA/CaJ mouse with ECM reveals that CD31 (PECAM-1) is predominantly present on the luminal surface of arteriolar and capillary endothelia (green), while CD14 is expressed on venous endothelia (red). The projection of a 3D stack also shows that the level of CD14 expression is somewhat patchy within a given PCV. B) Brain from a PbA-infected CBA/CaJ mouse that had been inoculated with Evans blue (red) and PE-conjugated anti-mouse CD14 (green). In contrast to arterioles (white star), PCV are lined with CD14-labeled endothelia. C) Brain from a PyXL-infected mouse with HP that had been inoculated with Evans blue and PE-conjugated anti-mouse CD14 (green). While monocytes exhibit a CD14-positive surface label (arrow), PCV endothelia are CD14-negative. Note that the vascular marker has leaked into the parenchyma of the mouse brain infected with PbA (A), but not with PyXL (B). A) Maximum projection of a Z-stack, B and C) snapshots from time series. Scale bars = 20 µm. (TIF) [file ppat.1002982.s005.tif]

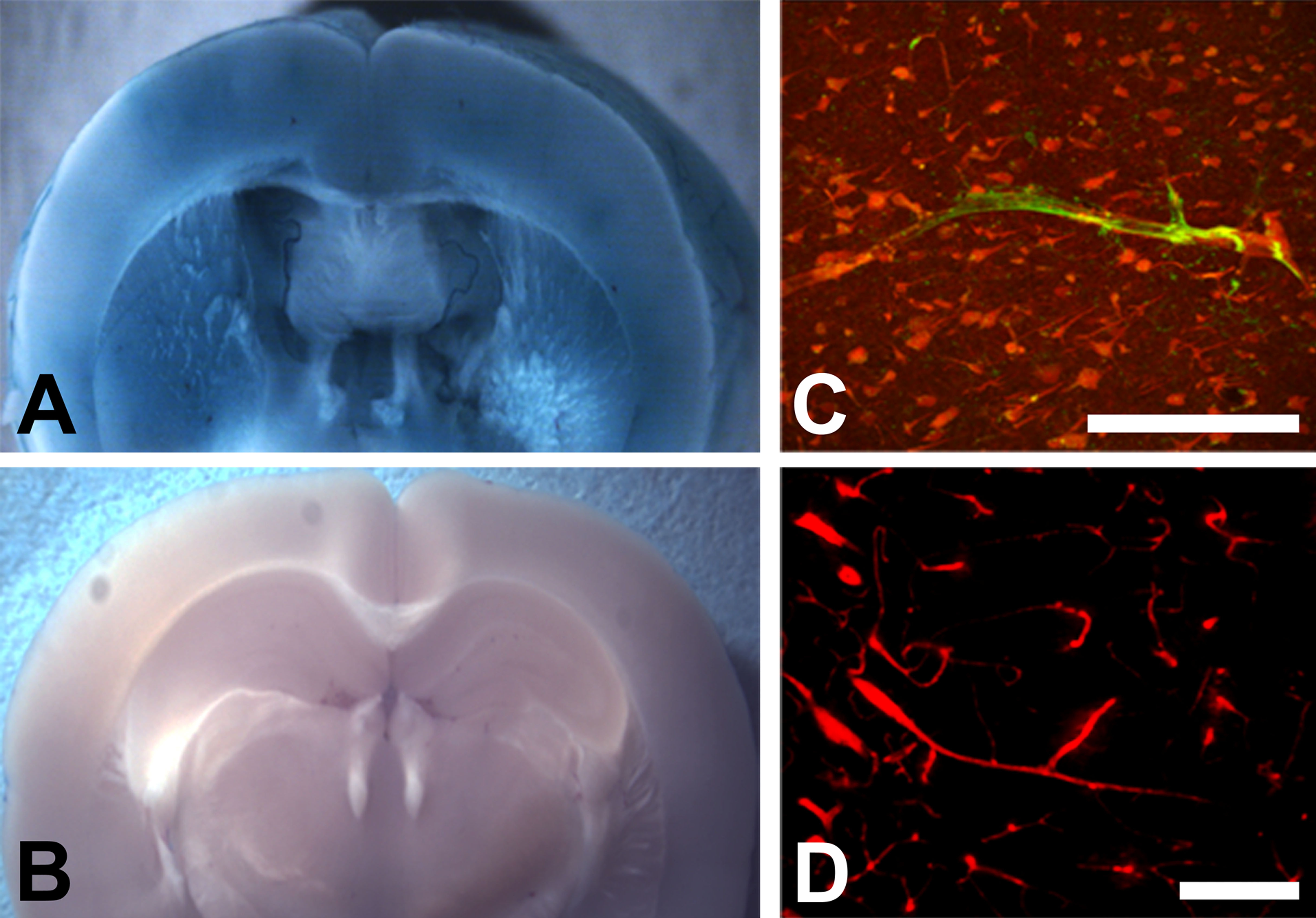

Supplement: Figure S6 — ECM is associated with vascular leakage throughout the brain. A and B) PbA-infected CBA/CaJ mice with neurological signs or uninfected mice were injected with Evans blue. Three hours later, the brains were removed and sectioned coronally at Bregma. A) The blue shade of the sectioned brain surface indicates that Evans blue has widely infiltrated the cerebral parenchyma. B) In contrast, brains from uninfected control mice appear pink and due to a lack of Evans blue leakage into the tissue. C and D) Upon ECM development, a PbA-infected CBA/CaJ mouse was injected with Evans blue and PE-conjugated anti-CD14. Three hours later, the brain was removed for preparation of coronal vibratome sections. C) The maximum projection of a confocal Z-stack, which was taken from the center of the gray matter, reveals Evans blue (red) has leaked from a PCV lined with CD14-positive endothelia (green) into the gray matter as evidenced by red-stained neurons. D) In contrast, neither Evans blue leakage nor CD14 labeling is detectable in a brain vibratome section from an uninfected control mouse. Scale bars = 20 µm. (TIF) [file ppat.1002982.s006.tif]

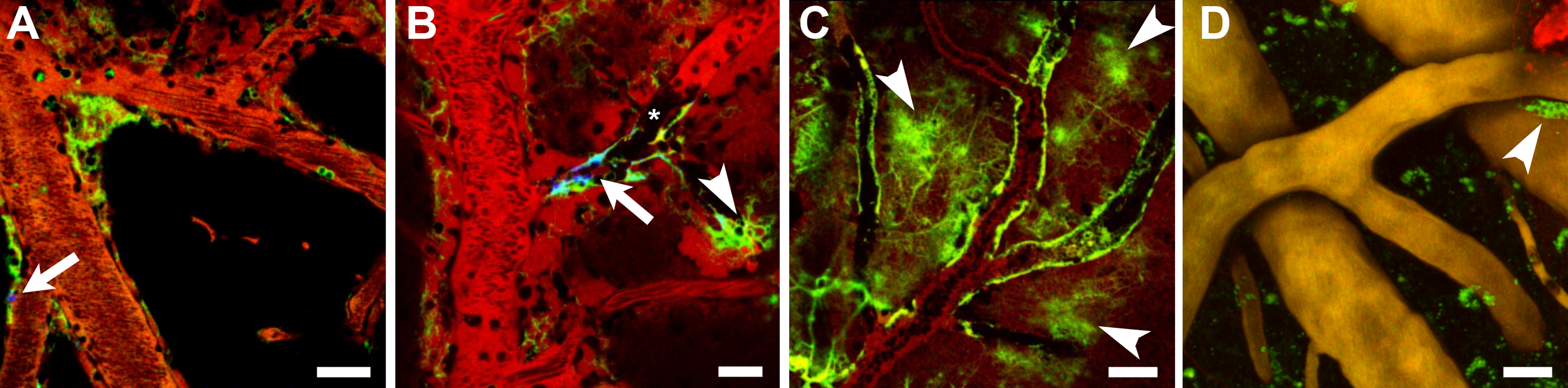

Supplement: Figure S7 — ECM is associated with perivascular fibrin deposition. A) PCV exhibit focal deposits of fibrin (or fibrinogen, green) within the PVS and occasional platelets (blue) in the absence of visible vascular injury. B) Rare sites of vascular injury are associated with aggregates of platelets (blue, arrow) and fibrin (green) deposits along the vascular wall, in the PVS, and in the parenchyma (arrowhead). The absence of luminal Evans blue (white star) indicates microvascular occlusion. C) Mechanical damage to the cortical microvasculature results in extensive fibrin leakage into the PVS and parenchyma (arrowheads). D) In uninfected control mice, fibrinogen remains uniformly distributed in the bloodstream and extravascular fibrin deposits were not detected. PbA-infected CBA/CaJ mice with ECM or uninfected control mice were inoculated with Alexa Fluor 488-conjugated human fibrinogen, platelets were detected with eFluor 401-conjugated anti-CD41. Bars = 50 µm. (TIF) [file ppat.1002982.s007.tif]

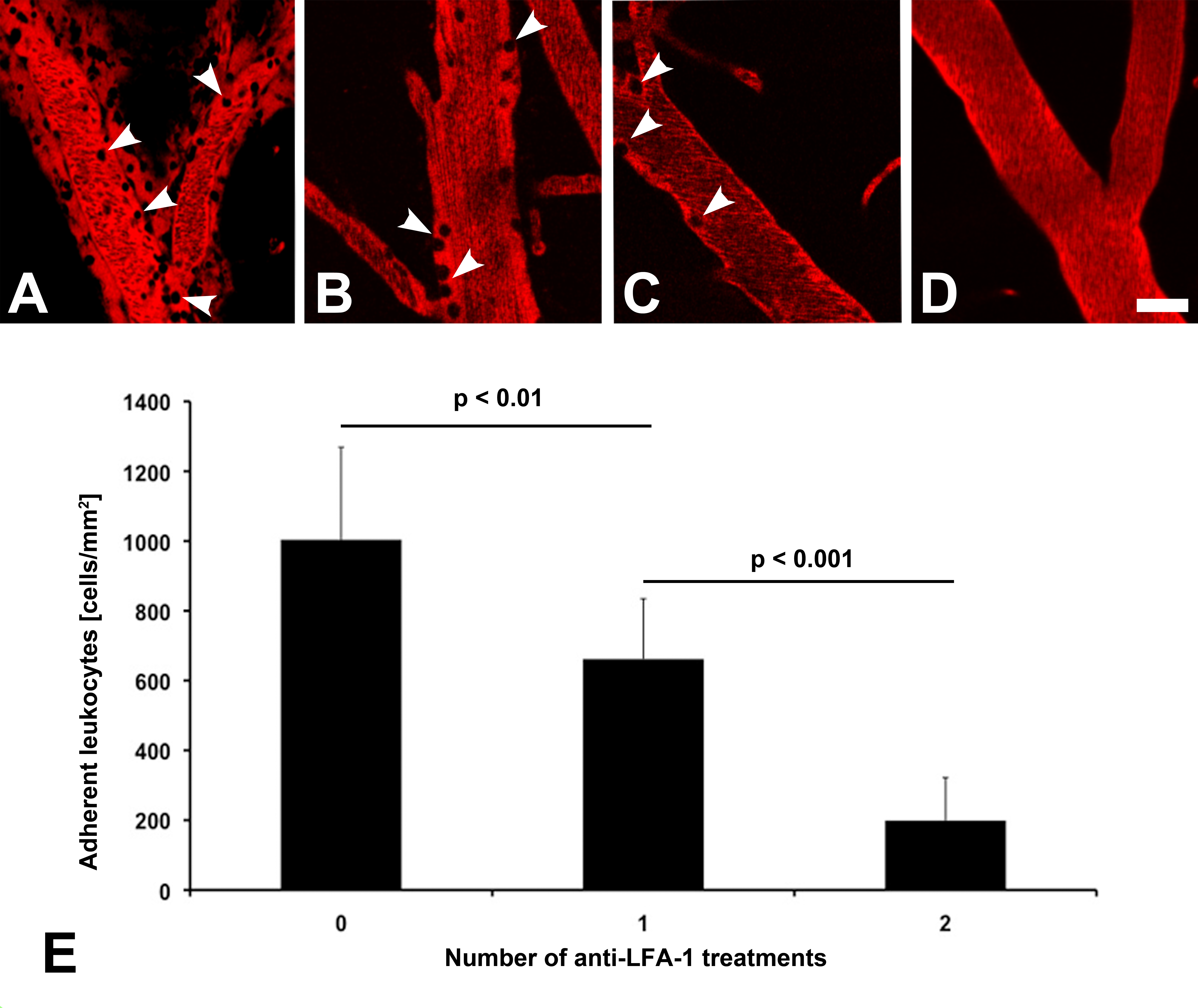

Supplement: Figure S8 — Anti-LFA-1 treatment reduces leukocyte recruitment. A) At the time of ECM (day 6), a PCV from a PbA-infected CBA mouse contains a large number of arrested leukocytes (arrowheads). Leukocytes exclude Evans blue and appear dark. Note the extensive leakage of the vascular marker into the PVS and surrounding tissue. B) Considerably fewer leukocytes are present in a PCV from a mouse that was imaged 2 days after a single anti-LFA-1 treatment on day 5. Note the absence of vascular leakage. C) Arrested leukocytes are rare and vascular leakage is absent after two anti-LFA-1 treatments on day 5 and 7. IVM was done 4 h after the second treatment. D) Cortical PCV from uninfected mice do not contain arrested leukocytes. Scale bar = 20 µm. E) Compared to untreated PbA-infected CBA/CaJ mice with ECM (0), one (1) and two (2) anti-LFA-1 treatments inhibit leukocyte arrest significantly in a dose-dependent manner. The data reflect the mean number of leukocytes/mm2 ± STD counted in 20–45 PCV depending on the experimental condition. Significance was determined with Student's t-test. (TIF) [file ppat.1002982.s008.tif]

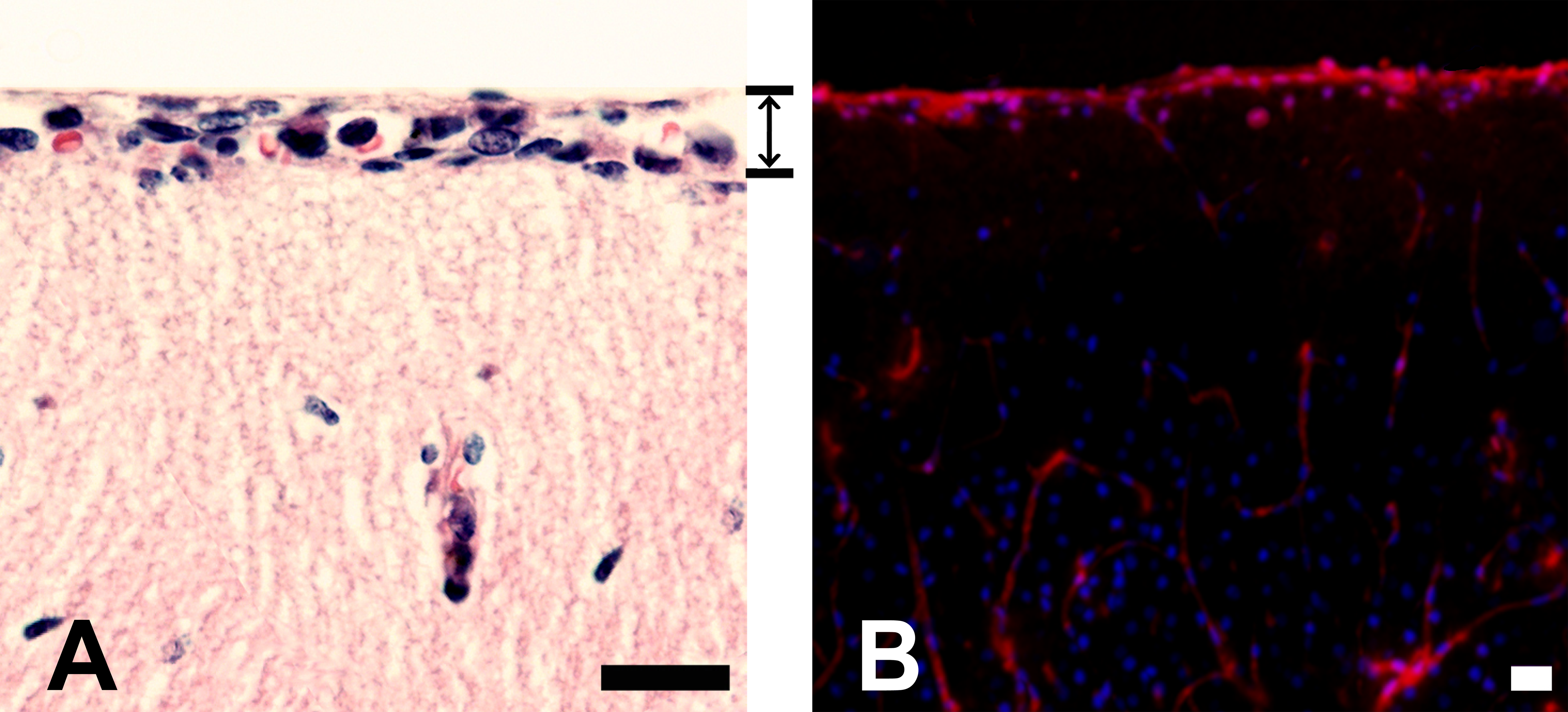

Supplement: Figure S9 — Thickness of murine meninges. A) H&E-stained sections of paraffin-embedded brain tissue show that the murine cerebral meninges measure 10–15 µm in diameter (double arrow). Note that compared to the underlying cortex, the meninges are highly vascularized and rich in nuclei. B) Coronal vibratome section of live brain tissue from a CBA/CaJ mouse that had been injected with the vascular marker Evans blue (red) and the nuclear stain Hoechst (blue). Note that compared to the cortical microvasculature, the meningeal blood vessels contain considerably larger amounts of Evans blue; confocal microscopy. Video S13. Scale bars = 20 µm. (TIF) [file ppat.1002982.s009.tif]

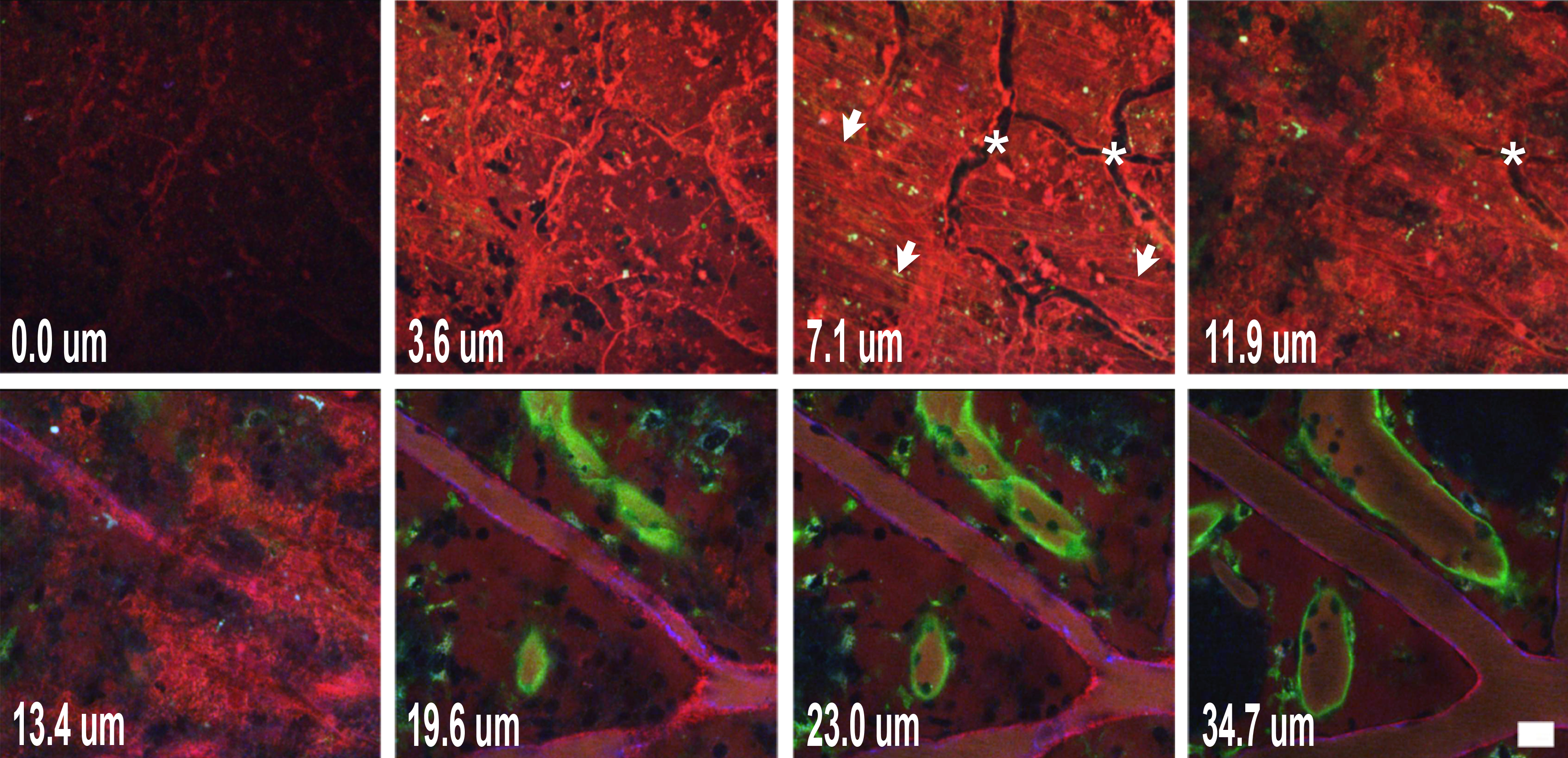

Supplement: Figure S10 — Confocal microscopy allows IVM of the cortical microvasculature. Individual frames from a confocal Z-stack, reaching from the Dura mater 50 µm into the cerebral cortex. Pial microvessels (white stars) are visible at a depth of 3.6 µm and 7.1 µm. Note the presence of meningeal collagen fibers (red autofluorescence, arrowheads) at a depth of 7.1 µm and 11.9 µm. Cortical arterioles (in vivo labeled with eFluor 405-conjugated anti-CD31, blue) and PCV (in vivo labeled with PE-conjugated anti-CD14, green) become visible at 13.4 µm and are fully in focus at a depth beyond 19.6 µm. Scale bar = 20 µm. Video S14. (TIF) [file ppat.1002982.s010.tif]
